# The STUB1-TPIT axis regulates the secretion of adrenocorticotrophic hormone in cushing disease

**DOI:** 10.1186/s12967-025-06960-y

**Published:** 2025-08-26

**Authors:** Fang Liu, Yanting Liu, Tao Zhang, Ning Huang, Desheng Chen, Xiaobin Wang, Jiangong Ma, Li Xue, Shaojian Lin, Zhe Bao Wu

**Affiliations:** 1 Department of Neurosurgery, Center of Pituitary Tumor, Ruijin Hospital, Shanghai Jiao Tong University School of Medicine, Shanghai, 200025 People’s Republic of China; 2 Department of Neurosurgery, First Affiliated Hospital of Wenzhou Medical University, Wenzhou, 325000 People’s Republic of China; 3 Department of Neurosurgery, Center for Immune-Related Diseases, Shanghai Institute of Immunology, Ruijin Hospital, Shanghai Jiao Tong University School of Medicine, Shanghai, 200025 People’s Republic of China; 4 The First Affiliated Hospital, Henan University, Kaifeng, 475004 People’s Republic of China; 5 Department of Biochemistry and Molecular Cell Biology, State Key Laboratory of Oncogenes and Related Genes, Shanghai Key Laboratory for Tumor Microenvironment and Inflammation, School of Medicine, Shanghai Jiao Tong University, Shanghai, 200025 People’s Republic of China; 6 Shanghai Center for Brain Science and Brain-Inspired Technology, Shanghai, 200022 People’s Republic of China

**Keywords:** Cushing's disease, Corticotroph adenoma, TPIT, STUB1, Ubiquitin-mediated degradation

## Abstract

**Background:**

Cushing’s disease (CD) is a clinical syndrome caused by excessive secretion of adrenocorticotropic hormone (ACTH) from a pituitary corticotroph adenoma, resulting in adrenal cortical hyperplasia and overproduction of cortisol. The T-box transcription factor (TPIT) is crucial for regulating ACTH secretion in pituitary corticotroph adenomas. This study aims to explore the ubiquitin-mediated degradation of TPIT and identify potential pharmaceutical agents for treating CD.

**Methods:**

The TPIT-interacting protein STUB1 was identified via mass spectrometry. The interaction between STUB1 and TPIT was confirmed using NanoBiT and GST pulldown assays. The expression of TPIT, pro-opiomelanocortin (POMC), and STUB1 was assessed by immunoblotting, dual-luciferase reporter assays, quantitative real-time PCR, RNA-sequencing, and immunohistochemistry. ACTH levels were measured by ELISA.

**Results:**

STIP1 Homology and U-Box Containing Protein 1 (STUB1) interacts with TPIT through its TPR domain and ubiquitinates multiple sites on TPIT via the U-box domain, leading to TPIT degradation. This degradation reduces POMC expression and ACTH secretion in AtT-20 cells. Additionally, STUB1 inhibits cell proliferation both in vitro and in vivo. Clinical investigations revealed that STUB1 expression is significantly lower in ACTH-secreting corticotroph adenomas than in silent corticotroph adenomas (SCAs). A negative correlation was observed between STUB1 and TPIT protein levels, as well as POMC expression. Furthermore, NanoBiT drug screening identified that Irbesartan and Lumiracoxib increased TPIT degradation, thereby reducing POMC expression and ACTH secretion.

**Conclusion:**

STUB1 is a promising therapeutic target for CD and drugs targeting the STUB1-TPIT complex may provide a potential treatment approach.

**Graphical Abstract:**

E3 ubiquitin ligase STUB1 interacts with TPIT, mediating its ubiquitination degradation, subsequently inhibiting POMC transcription and ATCH secretion. Irbesartan and Lumiracoxib can promote TPIT degradation and suppress ACTH secretion by enhancing the interaction between STUB1 and TPIT.
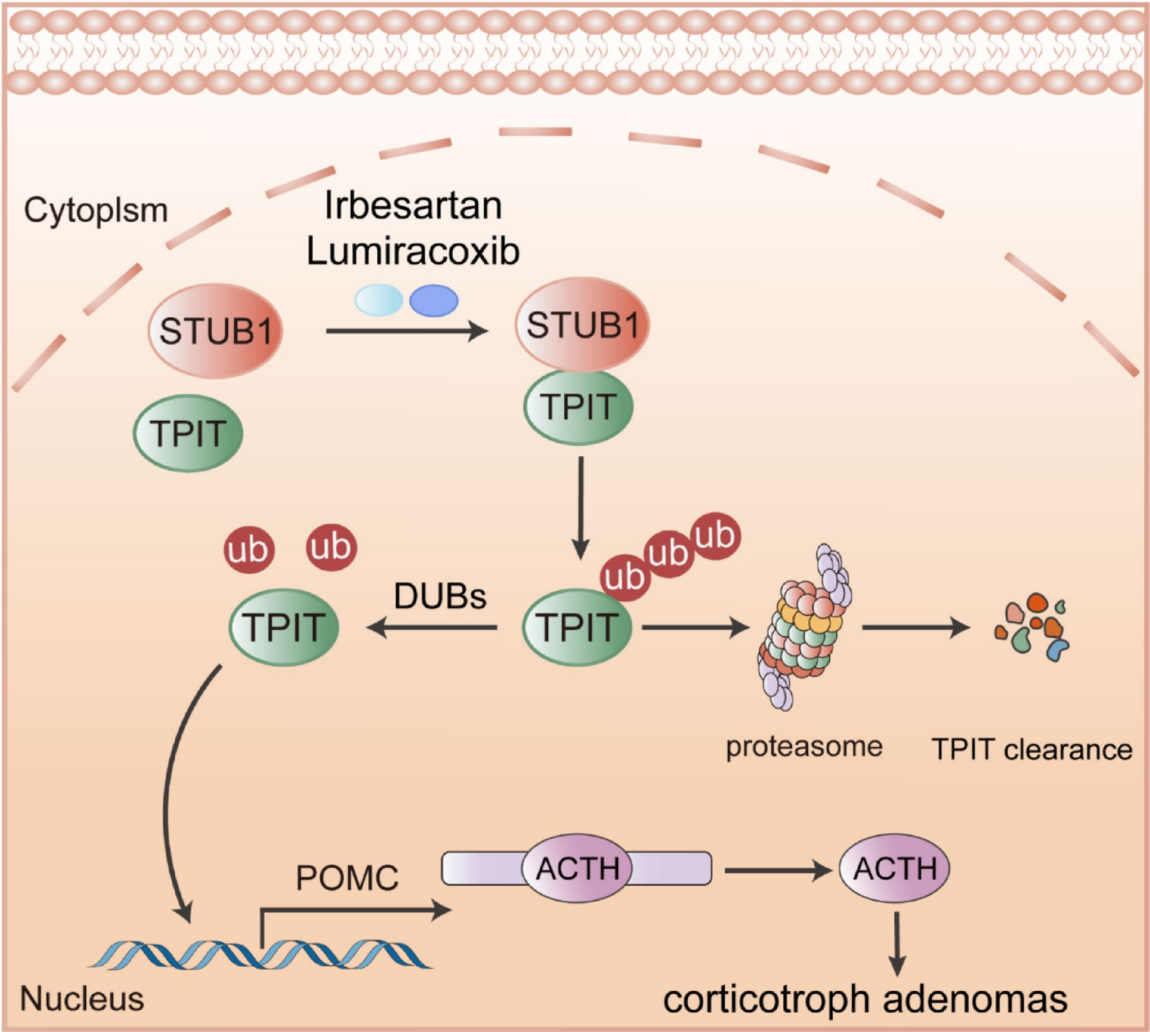

**Supplementary Information:**

The online version contains supplementary material available at 10.1186/s12967-025-06960-y.

## Introduction

Cushing's disease (CD) is a rare but clinically significant endocrine disorder characterized by excessive secretion of adrenocorticotropic hormone (ACTH) from a pituitary corticotroph adenoma. Corticotroph adenoma is characterized by expression of the transcription factor TPIT, which promotes the expression of the pro-opiomelanocortin (POMC) and ACTH secretion [1]. The excessive ACTH triggers a cascade of severe endocrine metabolic disorders and complications such as hypertension, diabetes mellitus, hyperlipidemia, osteoporosis, central obesity, and cardiovascular disease [2, 3]. These symptoms collectively impact the patient's quality of life and increase the risk of life-threatening complications like cardiovascular disease and infections. Cushing's disease is a highly challenging condition to manage, despite the availability of various treatment options, including surgery, medical therapy, and radiation therapy, which often fall short of achieving satisfactory outcomes. Elucidating the mechanisms underlying ACTH overproduction in Cushing's disease is essential to develop more effective treatments and address current clinical challenges.

Ubiquitination, the covalent attachment of ubiquitin to proteins, marks them for proteasomal degradation, maintaining cellular homeostasis [4]. Dysregulation of this process can cause aberrant protein accumulation, disrupt cellular functions, and drive tumor development and progression. In corticotroph adenomas, ubiquitination also plays a significant role [5]. For example, the mutations in the USP8 gene, present in 35–62% of ACTH adenomas, increase USP8 deubiquitinase activity, leading to enhanced epidermal growth factor receptor (EGFR) deubiquitination and subsequent elevation of ACTH secretion [6, 7]. Additionally, mutations in BRAF and USP48 also contribute to POMC transcription and ACTH production [8]. Our previous research showed that ubiquitination of TPIT by TRIM65 inhibits POMC transcription and ACTH production [9]. USP11 deubiquitinates and stabilizes TPIT, thereby promoting POMC transcription and ACTH secretion [10]. While these findings highlight the critical role of ubiquitination pathways in regulating ACTH secretion in corticotroph adenomas, a more detailed understanding of the underlying mechanisms driving ACTH overproduction is still needed. Moreover, the development of targeted therapies to modulate these pathways remains a significant unmet clinical need.

STIP1 Homology and U-Box Containing Protein 1 (STUB1) is a human gene encoding the C-terminus of HSC70-interacting protein (CHIP) [11], playing a crucial role in maintaining protein homeostasis by targeting misfolded or damaged proteins for proteasomal degradation [12]. Recent studies have highlighted the involvement of STUB1 in various human malignancies, as it regulates the stability and activity of several tumor-associated transcription factors, such as CRIP1, YAP1, HIF-1α, and c-Myc, thereby influencing tumor growth, invasion, metastasis, and angiogenesis [13–16]. Moreover, STUB1 has been shown to modulate the expression of tumor-secreted factors, such as cytokines, growth factors, and matrix metalloproteinases, by ubiquitinating and regulating transcription factors such as CSF2RB, NF-κB, and STAT3 [15, 17, 18]. Furthermore, STUB1 has been implicated in the modeling of the tumor immune microenvironment through its ability to regulate transcription factors that affect the secretory functions of both tumor and immune cells [19, 20]. Despite the extensive research on STUB1's functions in various biological processes and its emerging role in cancer biology, its specific involvement and potential mechanisms in corticotroph adenomas remain to be elucidated.

Here, we found that STUB1-mediated ubiquitination and subsequent degradation of TPIT led to an inhibition of ACTH secretion, becoming a potential therapeutic target for the management of Cushing's disease. The drugs Irbesartan or Lumiracoxib, which promoted the binding of STUB1 and TPIT, hold promise as viable avenues for the treatment of CD.

## Materials and methods

### Cell culture and reagents

A Mycoplasma Stain Assay kit (C0296, Beyotime, China) was used to test all cell lines for mycoplasma contamination prior to use. HEK-293T and AtT-20 cells were kindly provided by the Cell Bank, Chinese Academy of Sciences. In cell culture, the HEK293T cells (Passage number < 20) were cultivated in Dulbecco's Modified Eagle Medium (DMEM; Basal Media, L110KJ) supplemented with 10% (v/v) fetal bovine serum (FBS; Basal Media, S660JY). The AtT-20 cells (Passage number < 20) were cultured in Ham's F-12K (Kaighn’s) medium (Basal Media, L450KJ) supplemented with 2.5% FBS and 15% horse serum (Absin, abs989). All cell lines were maintained in a humidified atmosphere with 5% CO 2 at 37 ℃.

### Isolation and cultivation of pituitary tumor cells

Freshly obtained corticotroph tissues were rinsed with Hank's Balanced Salt Solution (HBSS, Gibco, 14,025,092) and cut into small pieces with a sterile scalpel. The fragments were digested in HBSS supplemented with 100 U/ml collagenase, type IV (Gibco, 17104019) at 37 ℃ for 6–8 h. Following digestion, the dispersed cells were filtered using a 100 μm cell strainer (Beyotime Biotechnology, FSTR100) and resuspended in ACK Lysing Buffer (Gibco, A1049201) for 5 min. The cells were subsequently resuspended in DMEM supplemented with 10% FBS and plated in a cell culture dish (BIOFIL, TCD010100). After 48 h of incubation, the floating tumor cells were utilized for subsequent experiments.

### Plasmids

Supplementary Table 1 provides additional details. The pLVshRNA-EGFP(2A)Puro cloning vector was used to generate the shRNAs targeting genes at various regions. The sequences of the shRNAs can be found in Supplementary Table 2.

### Transfection

To prepare the polyethyleneimine (PEI) transfection reagent, 1 g of Polyethylenimine Linear (PEI) with a molecular weight of 40,000 (Yeasen, 40816ES03) was precisely weighed and dissolved in 900 mL of ultrapure water within a 1 L glass beaker. The pH of the solution was carefully adjusted to a range of 6.80 to 6.90 using a sodium hydroxide solution. Subsequently, the solution was diluted to a final volume of 1 L. Finally, the solution was filtered through a 0.22 µm filter to obtain a clear and sterile 1 mg/mL PEI solution.

For cell transfection, HEK293T cells were seeded in culture dishes one day prior to transfection, with a target cell confluency of 70% to 90% at the time of transfection. For the PEI transfection procedure, a 1.5 mL centrifuge tube was prepared. Into this tube, 200 µL of Opti-MEM serum-free medium (Gibico, 31,985,070), 6 µg of plasmid DNA, and 18 µL of the PEI reagent were added, thereby maintaining a 1:3 mass/volume ratio of plasmid to PEI. The components were thoroughly mixed by vortexing for 30 s, followed by a brief centrifugation to collect the liquid at the bottom of the tube. The mixture was then incubated at room temperature for 15 min before being added dropwise to a 6 cm culture dish (BIOFIL, TCD010060). Subsequent experimental operations can be performed 24 to 48 h later.

### Mass spectrometry (MS)

Approximately 10 million primary corticotroph tumor cells were infected with Flag-TPIT adenovirus at a multiplicity of infection (MOI) of 10. The cells were lysed using a Triton X-100 lysis buffer (150 mM NaCl, 50 mM Tris, and 1% Triton X-100, pH 7.5), and the immunoprecipitation complex was isolated utilizing anti-DYKDDDDK affinity beads (Smart-lifesciences Biotechnology). The beads were washed four times with Triton X-100 lysis buffer and the proteins were eluted with elution buffer (8 M urea, 100 mM Tris, pH 8.0). The eluted proteins were subsequently identified using MS.

The 10 million HEK-293T cells were transfected with Flag-TPIT for 48 h, followed by lysis. Immunoprecipitation of Flag-TPIT and its bound proteins was performed using 100 μL DYKDDDDK affinity beads. The beads were then washed using lysis buffer and boiled in SDS-PAGE sample buffer for 5 min at 95 ℃. The interacting proteins in the gel were identified by immunoblotting and MS.

Proteomic data analysis was done by Shanghai Luming Biological Technology Co., Ltd. using an Easy-NLC1200 nano-HPLC system. Peptide samples were re-dissolved in Buffer A (0.1% formic acid aqueous solution) and separated on a 75 μm × 150 mm RP-C18 column at 300 nL/min. They were cleaned by a gradient with blank solvent for 30 min. The eluates were analyzed by Q-Exactive MS in DDA mode, scanning 300–1600 m/z. The 20 strongest fragment profiles were collected after each full scan using HCD with an NCE of 28 and dynamic exclusion of 25 s. MS1 resolution was 45,000 at m/z 200, AGC target 1E6, and max injection time 50 ms. MS2 resolution was 15,000, AGC target 1E5, and max injection time 50 ms.

### Co-immunoprecipitation (Co-IP)

The cells in 6 cm dish were lysed with 800 μL Triton X-100 lysis buffer containing a protease inhibitor cocktail and sonicated. 100 μL of the lysate was used as input, and the remaining 700 μL lysates were then incubated overnight with antibody (1–2 μg), followed by incubation with 30 μL rProtein A/G Beads (Smart-lifesciences Biotechnology, SA032025) for 1 h at room temperature. The immunoprecipitates were then washed five times with lysis buffer and analyzed by immunoblotting.

### Immunoblotting

The total proteins were extracted with Triton X-100 lysis buffer supplemented with protease inhibitors (NCM Biotech, P003) and subsequently sonicated with a Qsonica Q700 Sonicator. The protein concentrations were measured using a BCA protein assay kit (Yoche Biotech, YSD-500T), with absorbances at 562 nm measured using a Biotek 800 TS microplate reader. Aliquots of whole-cell lysates were mixed with SDS-PAGE loading buffer and separated on SDS-PAGE, followed by transfer onto polyvinylidene difluoride membranes (Millipore, ISEQ10100). The antibodies used for probing the blots are listed in Supplementary Table 3.

### Ubiquitination assay

HEK-293T cells were cultured in 6 cm plates and co-transfected with the total 6 μg plasmids for 24 h and then lysed with RIPA lysis buffer (50 mM Tris–HCl, 150 mM NaCl, 5 mM EDTA, 1% Triton X-100, 0.5% sodium pyrophosphate, 0.1% SDS, pH 7.4) containing a protease inhibitor cocktail. The whole-cell lysates were then incubated overnight with 30 μL anti-DYKDDDDK affinity beads. The beads were washed five times with lysis buffer and analyzed by immunoblotting using the specified antibodies.

### NanoBiT assay

HEK-293T cells were cultured in 6-well plates and transfected with plasmids containing LgBiT and SmBiT. The cells were lysed using cell lysis buffer (Vazyme, DL101–01), and centrifuged, and the proteins in the supernatant were quantified using a BCA protein assay kit. Equal volumes and amounts of protein were added to a white 96-well plate (Absin, abs7016), followed by the addition of the same volume of 20 μM luminescent substrate Furimazine (Topscience, T15359). The luminescence was measured using the BioTek Synergy Neo2 Hybrid Multimode Reader.

### Immunofluorescence microscopy

The AtT-20 cells were infected with lentivirus containing Flag-TPIT. Subsequently, 100,000 of the infected cells were seeded in a 12-well plate that had been pre-coated with Matrigel (Absin, abs9410). The cells were fixed with 4% formaldehyde (Beyotime, P0099) for 20 min and then washed with PBS (Servicebio, G4202). Following this, the cells were permeabilized using a cold 0.2% Triton X-100 solution for 15 min and incubated overnight at 4 ℃ with anti-STUB1 and Flag antibodies after blocking with 3% BSA. The cells were then incubated with secondary antibodies conjugated with Alexa 488 (Cell Signaling Technology, 4412S) or Alexa 555 (Cell Signaling Technology, 4409S) for 1 h at 37 ℃. Nuclei were stained using DAPI (Beyotime, C1002), and the stained sections were evaluated and imaged using a Zeiss LSM880 lightning confocal microscope (Carl Zeiss AG, Oberkochen, Germany).

### GST pulldown assay

The GST or His-tagged proteins were purified and GST pull-down assays were performed as previously described [21].

### Nuclear-cytoplasmic separation

Nuclear and cytoplasmic proteins were separated using the Nuclear and Cytoplasmic Protein Extraction Kit (Beyotime, P0027).

### In vitro ubiquitination assay

The GST-TPIT and STUB1-His proteins were purified, and subsequently subjected to an in vitro ubiquitination assay using the Ubiquitinylation kit (Enzo Life Sciences, BML-UW992).

### Stable cell lines

The HEK-293T cells were cultured in 10 cm plates. For transfection, 6 μg of psPAX2, 3 μg of pMD2.G, 9 μg of the targeting plasmid, and 54 μL of PEI transfection reagent were added to 1 mL of Opti-MEM medium. The mixture was vortexed and incubated at room temperature for 15 min before being added to the cell culture dishes. After 6-8 h of infection, the cell supernatant was removed and replaced with fresh cell culture medium. After 48 of transfection, media containing the virus were collected and filtered through 0.45μm nitrocellulose filters (Millipore, SLHV033RS). The virus was then concentrated using PEG8000 (5 × PEG8000: 150 mm NaCl; 25% PEG8000) and used to infect AtT-20 cells. After 6-8 h of infection, the cell supernatant containing the lentivirus was removed and replaced with fresh cell culture medium. After 48 h of infection, the medium was changed to one containing puromycin to select the infected cells. The stably transfected cells were selected using puromycin at 1 μg/ml for 1 week (Beyotime, ST551).

### Cycloheximide (CHX) Chase Assay

The 500,000 stable cells were seeded into a 6-well plate with 3 mL of culture medium. The cells were treated with 100 μg/ml CHX (ApexBio, A8244), collected at specified time intervals, and analyzed by immunoblotting.

### Dual-luciferase gene reporter assays

The pGL4.15 vector was utilized to create the luciferase reporter vector *Pomc*-Luc by inserting the mouse *Pomc* promoter sequence (–646 to + 65). HEK-293T cells were transfected with pGL4.15-*Pomc*-Luc, pRL-TK, Flag-*TPIT*, and Myc-*STUB1* (wild-type or mutants). The dual-luciferase reporter assay was conducted using the Dual Luciferase Reporter Assay Kit (Vazyme, DL101-01).

### Quantitative real-time PCR (qRT-PCR)

Prepare 100,000–200,000 cells, and total RNA was extracted from cells using a total RNA extraction kit (NCM Biotech, M5105). The concentration of RNA was determined using a DS-11 microphotometer. Subsequently, cDNA synthesis was carried out using the cDNA synthesis kit (ABclonal, RK20429). qRT-PCR amplification was performed using a SYBR Green qPCR kit (ABclonal, RK21203) in accordance with the manufacturer's instructions. The qRT-PCR was conducted using an ABI 7500 Real-Time PCR System (Applied Biosystems). The primer sequences used for qRT-PCR are listed in Supplementary Table 4.

### Luminescent cell viability assay

The numbers of AtT-20 cells were measured using a Beckman Coulter Z2 cell counter. Following this, a total of three thousand cells in 100 μL media were plated per well in white 96-well plates (Absin, abs7016). Subsequently, the cell viability was assessed using the CellCounting-Lite 2.0 Luminescent Cell Viability Assay (Vazyme, DD1101-03) with the BioTek Synergy Neo2 Hybrid Multimode Reader.

### Enzyme-linked immunosorbent assay (ELISA)

A total of 40,000 AtT-20 cells in 1 mL of culture medium were seeded into 12-well plates. The supernatants were collected after 24 h and then centrifuged at 4 ℃ at 2000 g for 20 min. The ACTH levels were measured by an ELISA kit (Immunoway, KE1520).

### Xenograft model

The Ethical Review Board at Shanghai Jiao Tong University School of Medicine's Ruijin Hospital approved of the study's animal experimentation. The protocols followed the Institutional Animal Care and Use Committee's (IACUC) guidelines. Four-week-old nu/nu female mice (The number of mice in each group was 5) were procured and maintained in a controlled specific pathogen-free environment. One million AtT-20 cells were combined with Matrigel (Yeasen, 40187) in a 100-μL volume and then injected subcutaneously. Xenograft tumor volumes were assessed by measuring two perpendicular diameters with calipers and individually calculated using the formula: volume = a × b^2^/2 (where a denotes length and b denotes width). The weights of the mice and the dimensions of the tumors were measured twice a week. Subsequently, the mice were euthanized, and the tumors were excised, measured, and photographed.

### Patient and tissue samples

Samples of pituitary adenomas (PAs) were obtained from patients undergoing surgical procedures at Ruijin Hospital between 2016 and 2023. The study received ethical approval from the Ethical Review Board of Ruijin Hospital, affiliated with Shanghai Jiao Tong University School of Medicine. All patients whose tumor tissues were utilized in this study provided written informed consent. Information on the patients is provided in Supplementary Table 5.

### RNA sequencing and GTEx data analysis

The raw mRNA read counts were normalized using Fragments Per Kilobase of exon model per Million mapped fragments (FPKM). Specifically, read counts from 107 normal pituitary samples obtained from the GTEx V8 database were normalized by FPKM. To address batch effects between our data and the GTEx normal data, the normalizeBetweenArrays function in the limma package in R, a tool commonly utilized for powering differential expression analyses in RNA-sequencing and microarray studies, was used. The information on the expression of the genes (*TPIT*, *POMC,* and *STUB1*) is shown in Supplementary Table 5.

### Immunohistochemistry (IHC)

The immunohistochemistry was performed as previously described [21]. For IHC staining of FFPE tumor tissue sections, dewax and rehydrate sections sequentially in dewaxing solutions (Ⅰ, Ⅱ, Ⅲ, 10 min each), anhydrous ethanol (Ⅰ, Ⅱ, Ⅲ, 5 min each), and distilled water (5 min). Perform antigen retrieval by boiling in sodium citrate buffer (pH 6.0) for 30 min using a microwave histoprocessor, then cool and wash in PBS (pH 7.4, 3 × 5 min). Block endogenous peroxidase with 3% hydrogen peroxide (25 min, RT, dark), then wash in PBS (3 × 5 min). Block with 3% BSA (or rabbit serum for goat primary antibodies, 30 min, RT). Incubate with primary antibody in PBS overnight at 4 ℃. Wash in PBS (3 × 5 min), then incubate with HRP-labeled secondary antibody (50 min, RT). Wash again in PBS (3 × 5 min), add DAB solution, and monitor under a microscope until brownish-yellow color appears, then rinse with tap water. Restain nuclei with Gill’s hematoxylin (3 min), wash, differentiate, and rinse with running water. Dehydrate in 75% alcohol, 85% alcohol, anhydrous ethanol (twice), n-butanol, and xylene (each for 5 min), then seal with mounting medium. Examine under a white light microscope.The expression of STUB1 and TPIT was assessed using the H-Score method [H-SCORE = ∑ (PI × I) = (percentage of cells with weak intensity × 1) + (percentage of cells with moderate intensity × 2) + (percentage of cells with strong intensity × 3)], using QuantCenter 2.3 software. The quantification of expression in each sample was conducted in 10 randomly selected fields (at a magnification of 400×) for each case, by two impartial observers who were unaware of the participants' medical characteristics. The information on the H-Scores (TPIT and STUB1) is shown in Supplementary Table 5.

### High-throughput Nanobit drug screening

The HEK-293T cells in a 10 cm dish were transfected with 3 µg of Smibt-TPIT and 3 µg of Lgibt-STUB1 plasmids for 24 h. Then, 25 μL of media containing 5000 cells were seeded into CulturPlate-384 (PerkinElmer, 6007680) using the Multidrop Combi (Thermo Fisher Scientific). Subsequently, an equal volume of media containing the luminescent substrate furimazine (20 μM) was added to each well of the 384-well plate. The luminescence emitted by Lgbit-Smbit was quantified using the Explorer high-throughput screening platform (PerkinElmer). The drugs, including 1913 compounds from the Approved Drug Library, were then introduced at final concentrations of 1 μM. The luminescence was measured at 15, 30, 60, and 120 min. The data can be found in Supplementary Table 6. This procedure was conducted at the National Center for Translational Medicine,·Shanghai.

### Surface plasmon resonance (SPR)

Surface Plasmon Resonance (SPR) binding assays were performed on a Biacore system (Cytiva) at 25 ℃. The ligand protein was immobilized onto a CM5 sensor chip via amine coupling: chip surface activation with NHS/EDC (10 μL/min, 420 s), protein coupling (30 and 50 μg/mL in pH 4.0 acetate buffer, 5 μL/min, 600 s + 300 s), and blocking with ethanolamine (10 μL/min, 420 s), achieving 8000 RU immobilization. Small molecule analytes (in PBST buffer) were injected using the LMW Kinetics method (flow rate: 30 μL/min; contact: 60 s; dissociation: 300 s) across flow cells 4 (reference) and 3 (ligand). Real-time binding responses were recorded in resonance units (RU).

### Statistical analysis

The data were analyzed using GraphPad Prism version 7 (GraphPad Software, La Jolla, CA, USA) and are presented as means ± standard deviation (SD). Statistical analyses encompassed two-tailed t-tests, one-way analysis of variance (ANOVA), two-way ANOVA, and Pearson's correlation coefficient (r). Statistical significance was denoted by *p* < 0.05 and is visually represented in the figures by one asterisk (*p* < 0.05), two asterisks (*p* < 0.01), or three asterisks (*p* < 0.001).

## Results

### The ubiquitin ligase STUB1 interacts with TPIT

**Fig. 1:**
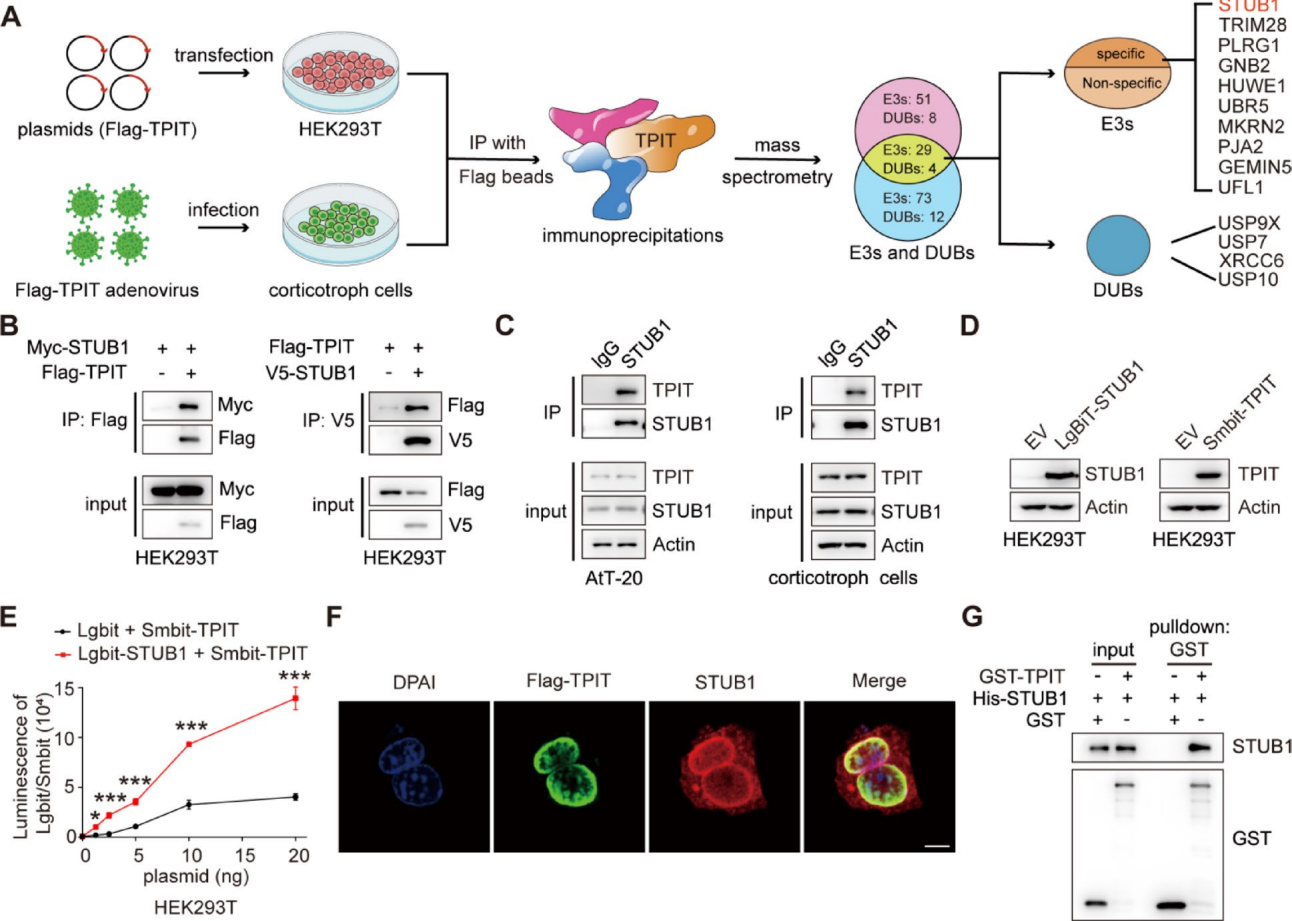
The ubiquitin ligase STUB1 interacts with TPIT.** A** Flowchart for the identification of ubiquitin ligases and deubiquitinases interacting with TPIT. TPIT and its interacting proteins were immunoprecipitated and identified by mass spectrometry. **B**,** C** Interaction of STUB1 with TPIT. External (B) and endogenous (C) Co-IP experiments were performed to detect the interaction between STUB1 and TPIT. **D**, **E** The NanoBiT assay was performed to detect the binding of STUB1 and TPIT. Data are presented as mean ± SD values. n = 3. ***p < 0.001. **F** Co-localization of Flag-TPIT and STUB1 in AtT20 cells. Scale bar, 5 μm. **G** The direct interaction between STUB1 and TPIT was determined by GST pulldown assay

The T-box transcription factor (TPIT) is crucial for regulating ACTH secretion in pituitary corticotroph adenomas [1, 22]. To elucidate the regulatory network of ubiquitination for TPIT, co-immunoprecipitation (Co-IP) and mass spectrometry analysis (MS) were employed to identify ubiquitin ligases and deubiquitinases associated with TPIT in HEK-293T and corticotropin cells. Ten potential ubiquitin ligases and four potential deubiquitinases were identified to interact with TPIT (Fig. 1A). The interactions between TPIT and the candidate ubiquitin ligases were further verified by Co-IP. It was found that STUB1, Tripartite motif-containing protein 28 (TRIM28), and PLRG1 interacted with TPIT, while the others did not (Fig. 1B, Supplementary Fig. 1A). Ubiquitin ligases can mediate the ubiquitination of substrates and promote their degradation through the proteasome pathway [23]. STUB1 and TRIM28 were identified as factors that promote the ubiquitination of TPIT, while PLRG1 did not exhibit this effect (Supplementary Fig. 1B). Additionally, both STUB1 and TRIM28 were found to decrease the protein levels of Flag-TPIT, with STUB1 showing greater efficacy in this regard (Supplementary Fig. 1C). Consequently, *STUB1* was chosen as a potential candidate gene for further investigation. Binding between endogenous STUB1 and TPIT was observed in AtT-20 and corticotropin cells by Co-IP analysis (Fig. 1C). Subsequent NanoBiT assays using Smbit-TPIT and Lgbit-STUB1 also confirmed the interaction between STUB1 and TPIT (Fig. 1D, E). Furthermore, immunofluorescence analysis demonstrated the co-localization of TPIT and STUB1 proteins in AtT-20 cells (Fig. 1F). In vitro experiments revealed that purified His-STUB1 directly interacted with the glutathione S-transferase (GST)-TPIT fusion protein, but not with GST alone (Fig. 1G).

Thus, these data indicated that the ubiquitin ligase STUB1 interacts directly with TPIT.

### STUB1 enhances the ubiquitination of TPIT

**Fig. 2:**
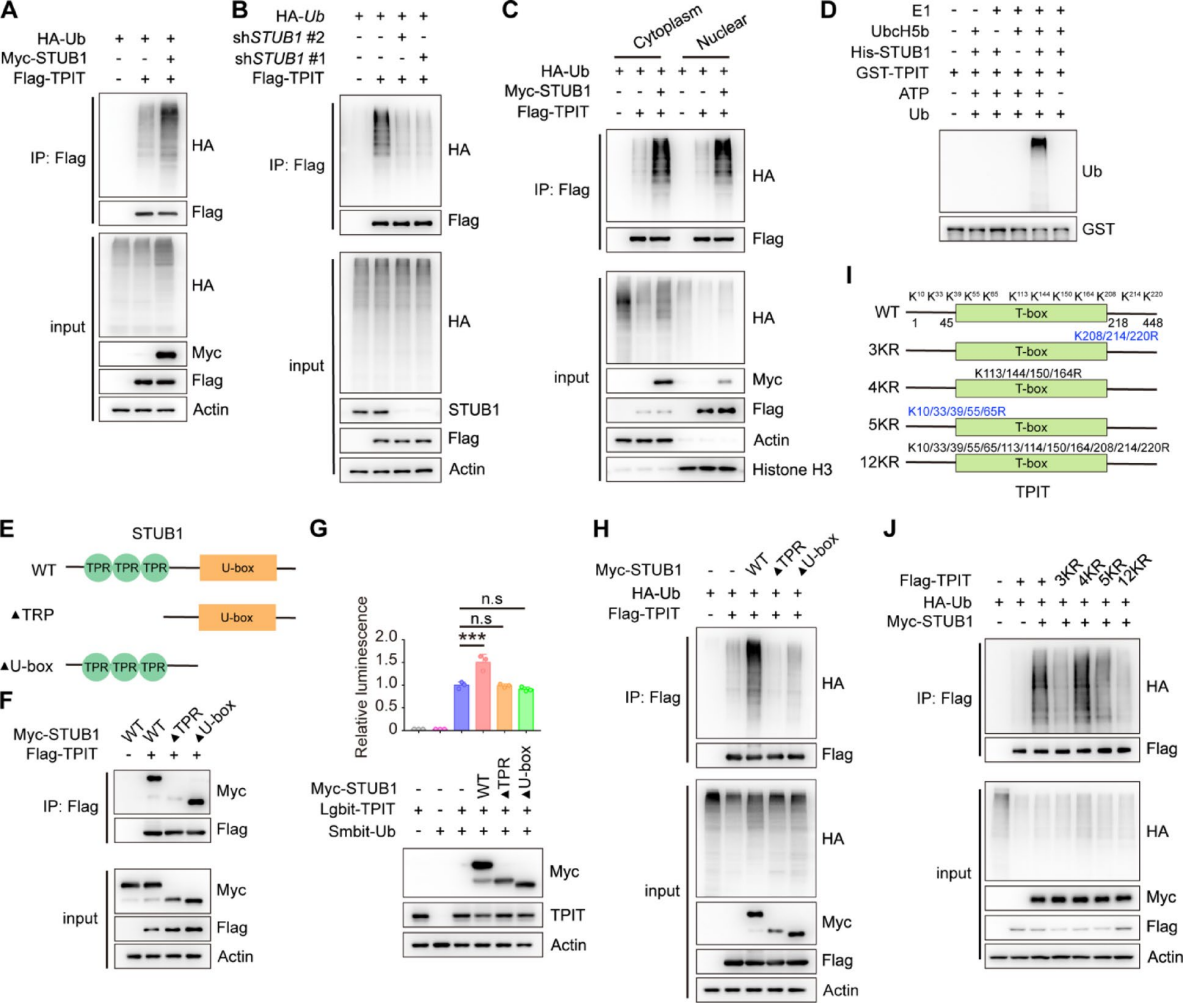
STUB1 enhances the ubiquitination of TPIT. **A** STUB1 promoted the ubiquitination of TPIT. HEK293T cells were transfected with the indicated plasmids, and cell lysates were analyzed by ubiquitination assays. **B** Knockdown of *STUB1* reduced TPIT ubiquitination. **C** TPIT ubiquitination was determined by nuclear-cytoplasmic fractionation. **D** In vitro ubiquitination assays showing ubiquitination of purified TPIT by purified STUB1. **E** Schematic showing wild-type STUB1 and its truncated forms. **F** STUB1 lacking the TPR domain was unable to interact with TPIT. The interaction between Flag-TPIT and Myc-tagged STUB1 or it truncated mutants was detected by Co-IP. **G**,** H** NanoBiT (G) and ubiquitination assays (H) showed that STUB1 lacking the TPR or U-box domains failed to ubiquitinate TPIT. Data are presented as mean ± SD values. n = 3. ***p < 0.001. **I** Schematic showing wild-type TPIT and its mutants. Mutation oflysine (K) to arginine (R). **J** Ubiquitination assays showed reduced ubiquitination of TPIT with the 3KR, 5KR, and 12KR mutations by STUB1

STUB1 is an E3 ubiquitin ligase that mediates the ubiquitination of various transcription factors, including CRIP1, YAP1, HIF-1α, and c-Myc [13–15]. Likewise, STUB1 increased the ubiquitination of TPIT, while knockdown of STUB1 decreased TPIT ubiquitination (Fig. 2A, B, Supplementary Fig. 2A, B). STUB1 is also known to mediate ubiquitin-dependent degradation of target proteins from membranes, the cytoplasm, and the nucleus [24]. The subcellular localization of STUB1 in HEK-293T cells was predominantly observed in the cytoplasm, with some presence in the nucleus (Supplementary Fig. 2C). Subsequent nuclear-cytoplasmic fractionation experiments revealed that STUB1 facilitated TPIT ubiquitination in both cellular compartments (Fig. 2C). Additionally, in vitro ubiquitination assay also confirmed that STUB1 promoted TPIT ubiquitination (Fig. 2D). Further exploration of the STUB1-TPIT interaction region was conducted through Co-IP experiments, which indicated that STUB1 lacking the TPR domain were unable to interact with TPIT (Fig. 2E, F). NanoBiT and ubiquitination assays demonstrated that STUB1, lacking the TPR or U-box domains, was unable to ubiquitinate TPIT (Fig. 2G, H). To further elucidate the specific lysine (K) sites on TPIT targeted for ubiquitination by STUB1, we generated various TPIT mutants (3KR, 4KR, 5KR, and 12KR) as outlined in a previous study [11]. Subsequent ubiquitination assays revealed that TPIT mutants with the 3KR, 5KR, and 12KR mutations exhibited decreased ubiquitination by STUB1 (Fig. 2I-J).

In summary, our findings suggest that STUB1 interacts with TPIT via the TPR domain and employs the U-box domain to ubiquitinate multiple sites on TPIT.

### STUB1 mediates proteasomal degradation of TPIT

**Fig. 3:**
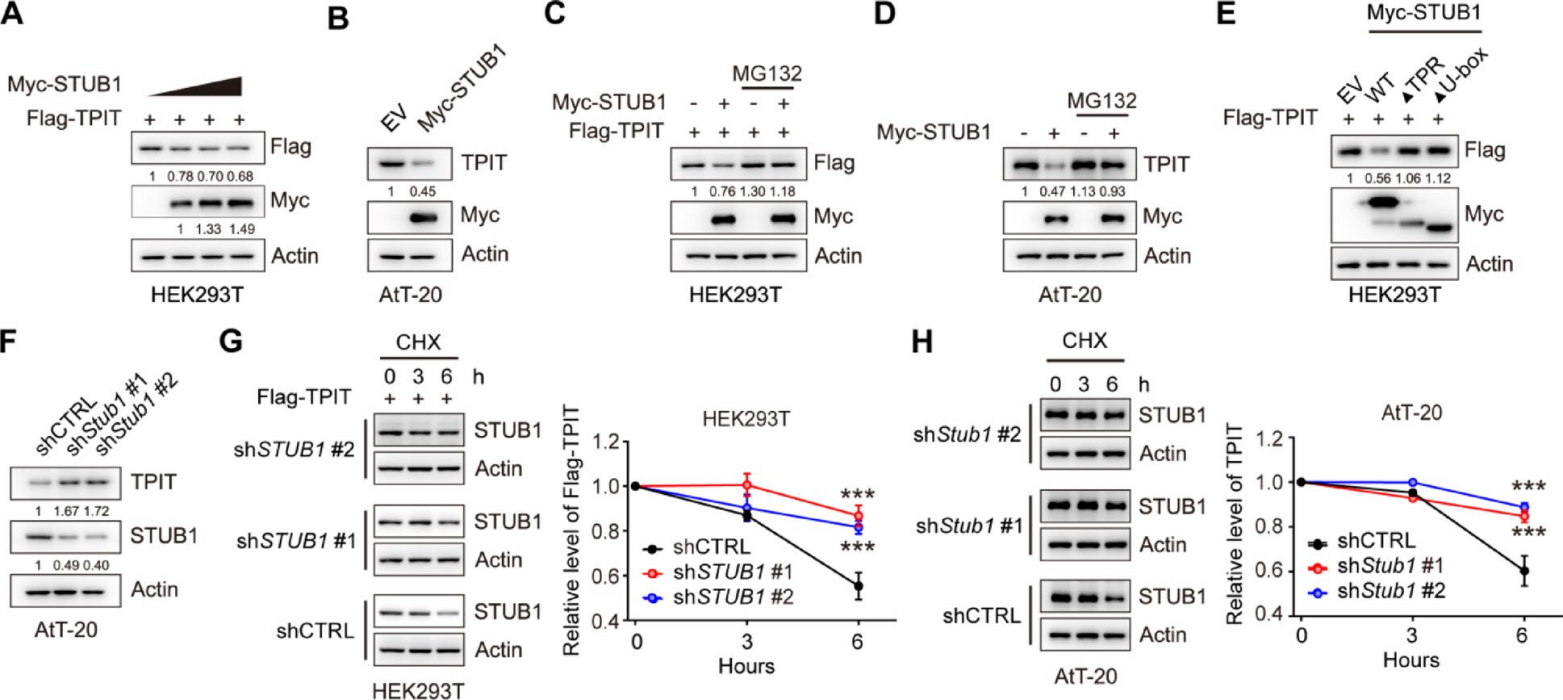
STUB1 mediates proteasomal degradation of TPIT. **A**, **B** Overexpression of *STUB1* reduced the protein levels of TPIT in HEK-293T (A) and AtT-20 (B) cells. **C**,** D** Proteasome inhibitor MG132 (20 μM) prevented STUB1-mediated degradation of TPIT in HEK-293T (C) and AtT-20 (D) cells. **E** Immunoblotting showed that STUB1 lacking the TPR or U-box domains failed to reduce TPIT protein levels in HEK-293T cells. **F** Knockdown of *Stub1* increased TPIT protein levels in AtT-20 cells. Stable AtT-20 cells with *Stub1* knockdown were immunoblotted. **G**, **H** CHX chase assays showed that knockdown of *STUB1* extend the half-life of TPIT in HEK-293T (G) and AtT-20 (H) cells. The cells were treated with CHX (100 μg/mL) for 0, 3, or 6 h. The relative ratio of TPIT/actin was determined by ImageJ. Data are presented as mean ± SD values. n.s., not significant; n = 3. ***p < 0.001

In order to investigate the regulatory effect of STUB1 on TPIT expression, STUB1 was overexpressed in HEK-293T and AtT-20 cells. The results demonstrated that the overexpression of STUB1 led to a decrease in TPIT protein levels in both cell lines (Fig. 3A, B, Supplementary Fig. 3A). However, the inhibition of proteasomal activity with the proteasome inhibitor MG132 reversed this reduction in TPIT protein levels induced by STUB1 in HEK293T and AtT-20 cells, suggesting that the downregulation of TPIT by STUB1 occurs through the proteasomal pathway (Fig. 3C, D). Moreover, the absence of the TPR or U-box domains in STUB1 resulted in the inability to decrease TPIT protein levels (Fig. 3E). Additionally, knockdown of *Stub1* led to an increase in TPIT protein levels in AtT-20 cells (Fig. 3F, Supplementary Fig. 3B). To further investigate the role of STUB1 in regulating TPIT stability, cycloheximide (CHX) chase assays were conducted, revealing that depletion of *STUB1* extended the half-life of Flag-TPIT in HEK-293T cells (Fig. 3G). Similarly, knockdown of *Stub1* extended the half-life of TPIT in AtT-20 cells (Fig. 3H).

These findings collectively demonstrate that STUB1 promotes the proteasomal degradation of TPIT.

### STUB1 reduces expression of POMC and secretion of ACTH

**Fig. 4:**
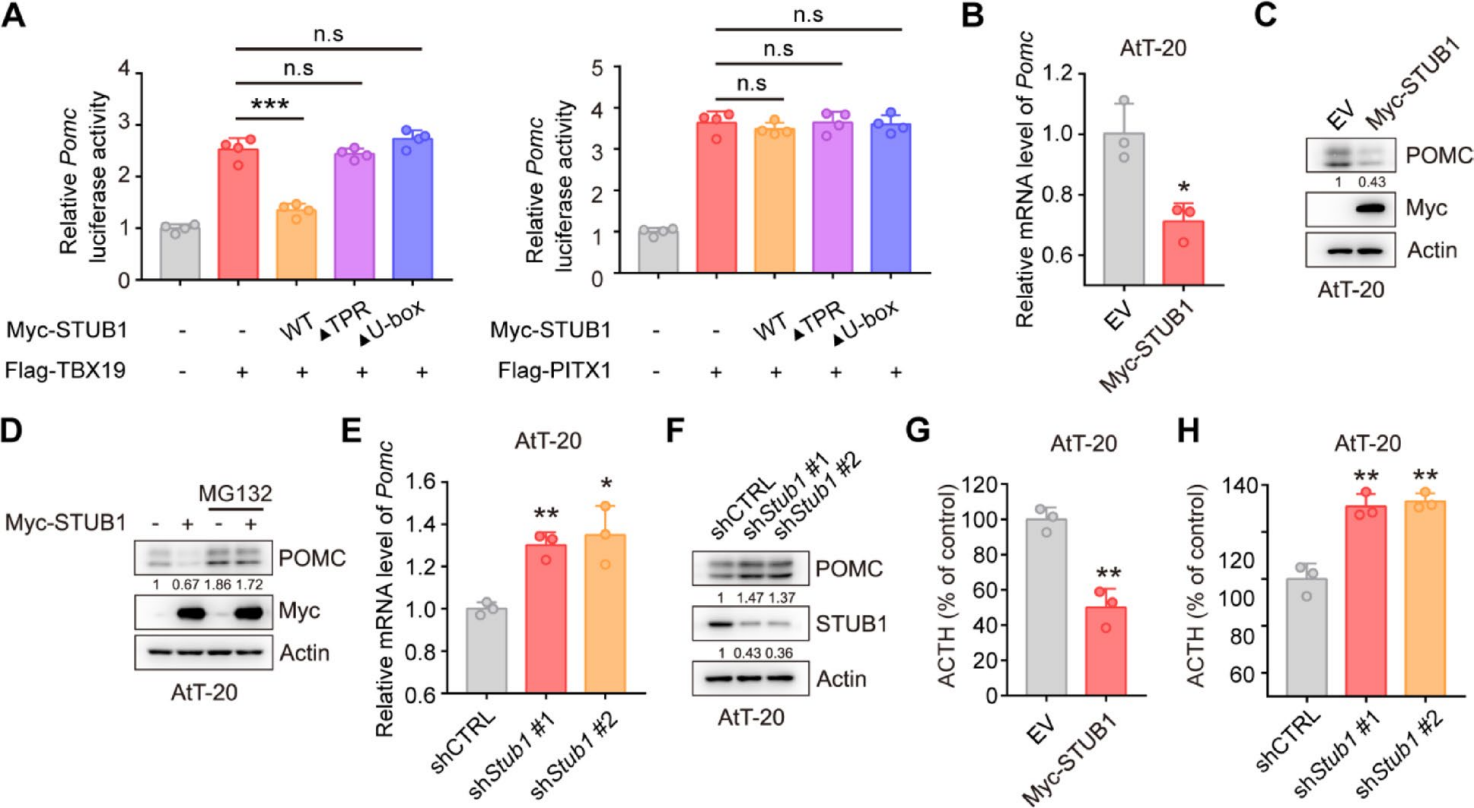
STUB1 reduces the expression of *POMC* and secretion of ACTH. **A** Dual-luciferase reporter assay demonstrated that STUB1 suppressed the transcription of *Pomc* mediated by TPIT. Data are presented as mean ± SD values. n.s., not significant; n = 3. ***p < 0.001. **B** qPCR showed that STUB1 suppressed the transcription of *Pomc* in AtT-20 cells. Data are presented as mean ± SD values. n = 3. ***p < 0.001. **C**, **D** STUB1 reduced POMC protein levels (C) with this effect reversed by the proteasomal inhibitor MG132 (D) in AtT-20 cells. **E**,** F** Knockdown of *Stub1* enhanced the mRNA (E) and protein (F) expression of POMC in AtT-20 cells. Data are presented as mean ± SD values. ***p < 0.001. **G**,** H** STUB1 regulated the secretion of ACTH in AtT-20 cells. The secretion of ACTH from stable AtT-20 cells with *Stub1* overexpression (G) or knockdown (H) was measured by ELISA. Data are presented as mean ± SD values. n = 3. ***p < 0.001

TPIT and PITX1 were identified as promoters of *Pomc* gene transcription [25]. Subsequent investigation into the impact of STUB1 on *Pomc* transcription utilized dual-luciferase reporter assays, revealing that STUB1 inhibited TPIT-mediated *Pomc* transcription while having no effect on PITX1-mediated *Pomc* transcription (Fig. 4A). Additionally, qPCR analysis demonstrated that STUB1 suppressed *Pomc* transcription without affecting *Tpit* transcription in AtT-20 cells (Fig. 4B, Supplementary Fig. 3C). In order to examine the impact of STUB1 on POMC protein levels, immunoblotting was conducted, revealing that STUB1 decreased the levels of POMC protein, with this effect being reversed by the proteasomal inhibitor MG132 (Fig. 4C, D). Additionally, knockdown of *Stub1* increased *Pomc* transcription without affecting *Tpit* transcription in AtT-20 cells (Fig. 4E, Supplementary Fig. 3D). Knockdown of *Stub1* resulted in elevated POMC protein expression in AtT-20 cells (Fig. 4F). Additionally, the impact of STUB1 on proliferation in AtT-20 cells was investigated, revealing that overexpression of STUB1 suppressed cell proliferation, whereas knockdown of *Stub1* enhanced cell proliferation (Supplementary Fig. 4A, B). Importantly, the effects of STUB1 on the ACTH secretion were then evaluated using enzyme-linked immunosorbent assay (ELISA), showing that overexpression of STUB1 inhibited the secretion of ACTH, while *Stub1* knockdown increased the secretion of ACTH in AtT-20 cells (Fig. 4G, H).

Collectively, these data indicated that STUB1 downregulates the expression of POMC and inhibits the secretion of ACTH.

### STUB1 inhibits TPIT and POMC expression, as well as ACTH secretion in vivo

**Fig. 5:**
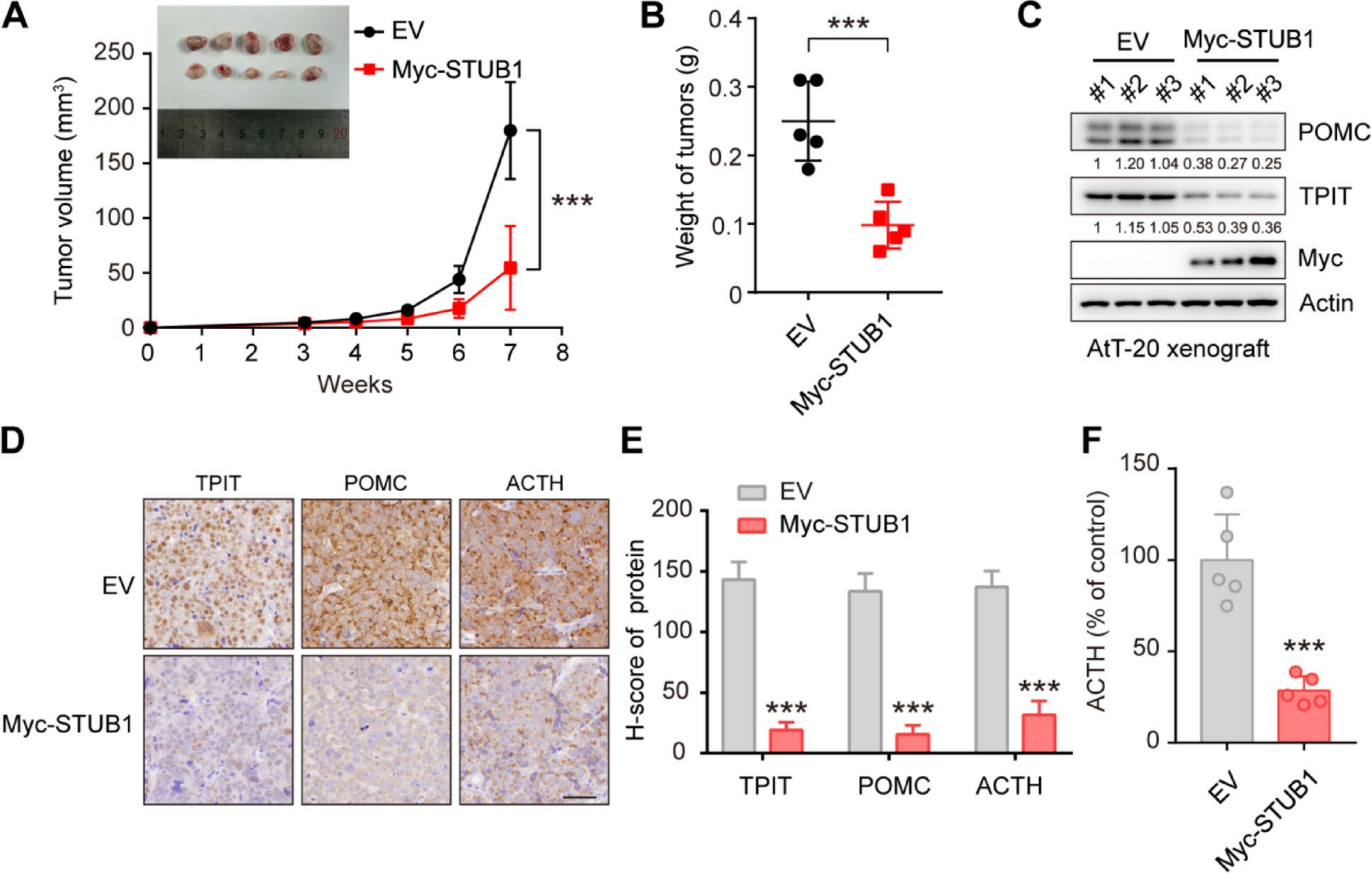
STUB1 inhibits TPIT and POMC expression, as well as ACTH secretion in vivo. **A**, **B** STUB1 overexpression inhibited tumor growth in vivo. The TRIM21-overexpression or control AtT-20 cells were implanted subcutaneously in nude mice. Xenograft tumors were imaged (A), measured (A) and weighed (B). Data are presented as mean ± SD values. n = 5. ***p < 0.001. **(C)** STUB1 overexpression inhibited TPIT and POMC expression in vivo. The STUB1-overexpressing and control xenograft tumors were harvested and immunoblotted. **D**,** E** Immunohistochemistry of xenograft tumors. Representative IHC images of proteins in STUB1-overexpressing and control xenograft tumors. Scale bar, 50 μm. Semi-quantitative immunohistochemical analysis of proteins. Data are presented as mean ± SD values. **F** STUB1 inhibited the secretion of ACTH in vivo. The secretion of ACTH from nude mice with xenograft tumors was measured by ELISA. Data are presented as mean ± SD values. n = 5. ***p < 0.001

To investigate the impact of STUB1 on TPIT, POMC, and ACTH in vivo, STUB1-overexpressing or control AtT-20 cells were implanted into nude mice. The results showed that STUB1 overexpression significantly inhibited xenograft tumor growth compared to the control group (Fig. 5A, B). Furthermore, immunoblotting analysis revealed that STUB1 overexpression led to a significant reduction in the protein levels of TPIT and POMC (Fig. 5C). Consistently, immunohistochemistry analysis demonstrated that STUB1 overexpression resulted in reduced levels of POMC (Fig. 5D, E). Most importantly, STUB1 overexpression significantly inhibited the secretion of ACTH in vivo (Fig. 5F).

Taken together, these findings suggest that STUB1 acts to inhibit cell proliferation, decrease the expression of TPIT and POMC, and reduce ACTH secretion in vivo.

### STUB1 is decreased in ACTH-secreting corticotroph adenoma and negatively correlated with TPIT protein

**Fig. 6:**
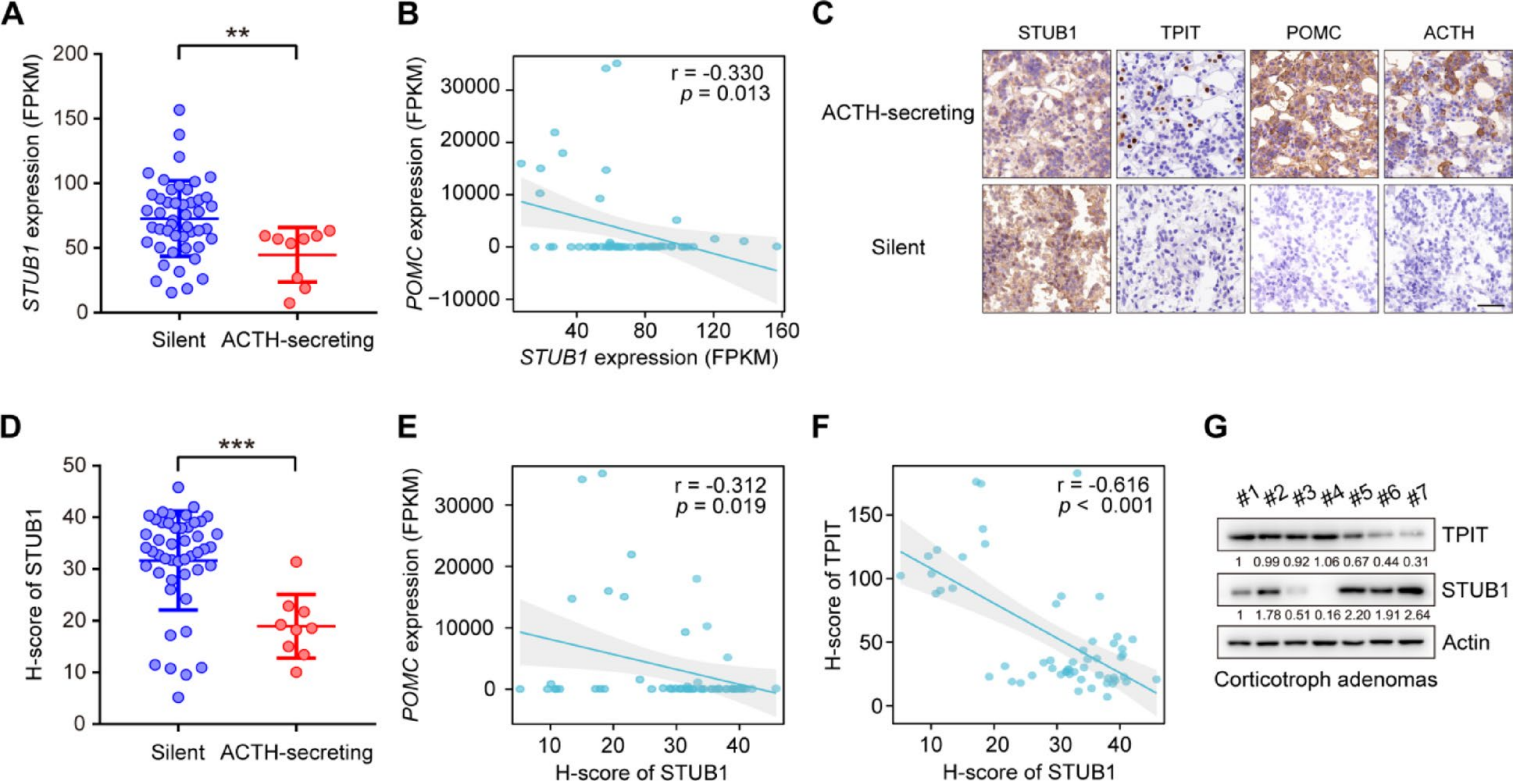
STUB1 is decreased in ACTH-secreting corticotroph adenoma and negatively correlated with TPIT protein. **A** *STUB1* expression was decreased in ACTH-secreting corticotroph adenoma. The expression of *STUB1* in functioning (n = 9) and silent (n = 47) corticotroph adenomas was analyzed by RNA-seq. Data are presented as mean ± SD values. **p < 0.01. **B** Correlation between mRNA expression of *STUB1* and *POMC* in corticotroph adenomas. **C** Representative immunohistochemical images showing expression of STUB1, TPIT, POMC, and ACTH in functioning and silent corticotroph adenomas. **D** Semi-quantitative immunohistochemical analysis of nuclear-localized STUB1 in functioning and silent corticotroph adenomas. **E** Correlation between nuclear-localized STUB1 protein and mRNA levels of *POMC* in corticotroph adenomas. **F**, **G** Correlation between STUB1 and TPIT protein levels in corticotroph adenomas, shown by immunohistochemistry (F) and immunoblotting (G)

To explore the role of STUB1 in the corticotroph pituitary, its mRNA expression in human normal pituitary and corticotroph adenomas was analyzed. Compared to normal pituitary glands, the expression of *STUB1* was found to be downregulated in corticotroph adenomas (Supplementary Fig. 5A). Additionally, corticotroph adenomas are divided into ACTH-secreting corticotroph adenomas and silent corticotroph adenomas (SCAs) [2]. SCAs demonstrate immunopositivity for ACTH in the absence of biochemical and clinical signs of hypercortisolism [26]. Compared to SCAs, *STUB1* was downregulated in ACTH-secreting corticotroph adenomas (Fig. 6A). Additionally, the relationships between the expression of STUB1, TPIT, and POMC were then analyzed. The mRNA expression of *STUB1* was observed to be negatively correlated with the mRNA expression of *POMC*, while no significant association was seen with the mRNA levels of *TPIT* in 56 corticotroph adenomas (Fig. 6B, Supplementary Fig. 5B). Furthermore, immunohistochemistry (IHC) showed that nuclear-localized STUB1 was downregulated in ACTH-secreting corticotroph adenomas, accompanied by increased levels of TPIT, POMC, and ACTH compared to SCAs (Fig. 6C). Then a semi-quantitative immunohistochemical analysis of nuclear-localized STUB1 was performed, demonstrating that STUB1 protein levels were also downregulated in ACTH-secreting corticotroph adenoma compared to SCAs (Fig. 6D). The nuclear-localized STUB1 was negatively correlated with the mRNA expression of *POMC*, but not significantly associated with *TPIT* mRNA levels in corticotroph adenomas (Fig. 6E, Supplementary Fig. 5C). The relationship between the protein expression of STUB1 and TPIT was then analyzed. Immunohistochemical analysis showed that nuclear-localized STUB1 was negatively correlated with TPIT protein, which was verified by immunoblotting (Fig. 6F, G).

Taken together, the findings indicate that STUB1 is decreased in ACTH-secreting corticotroph adenomas and is negatively correlated with *POMC* expression and TPIT protein.

### Irbesartan and Lumiracoxib enhance the interaction between STUB1 and TPIT, reducing POMC expression and ACTH secretion

**Fig. 7:**
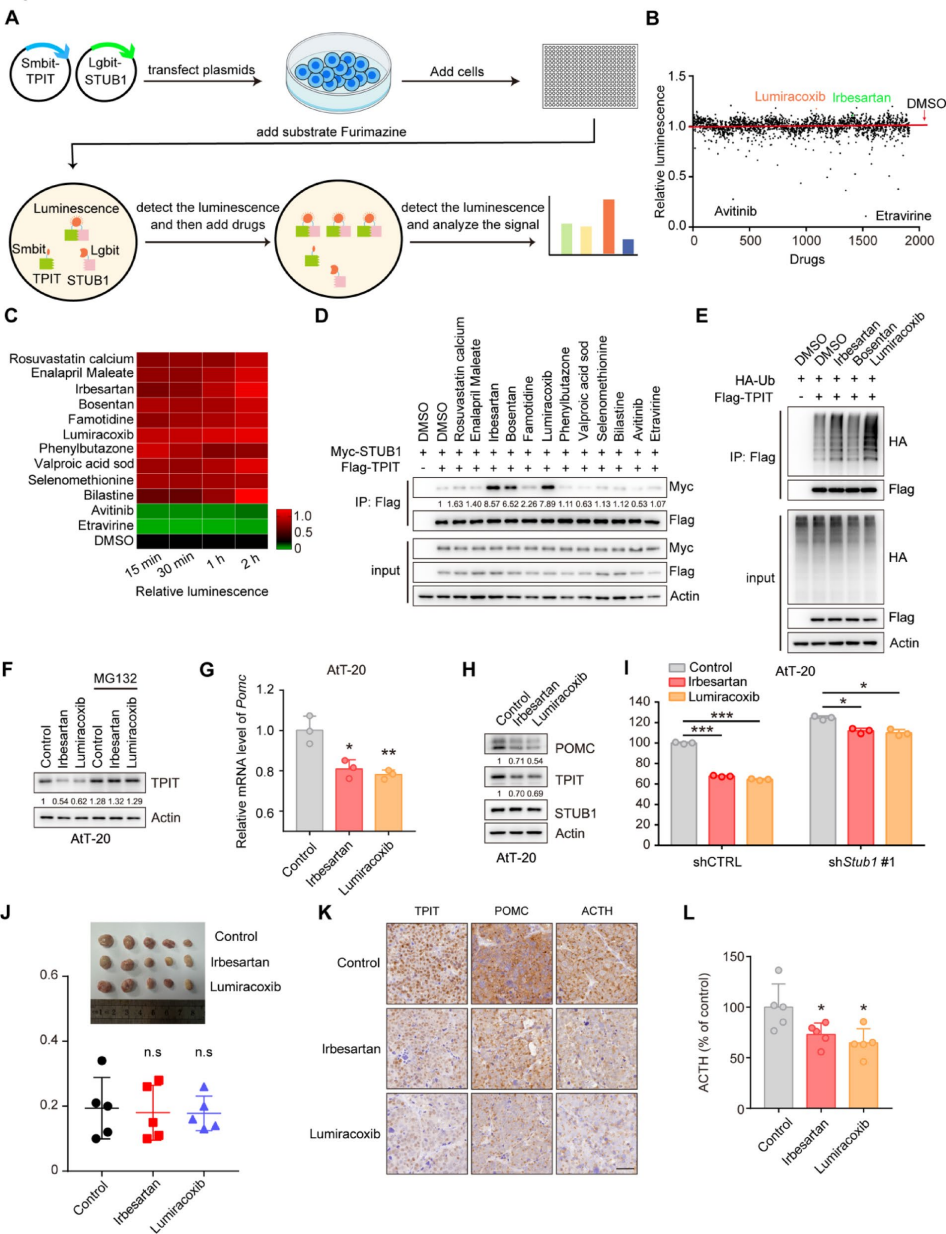
Irbesartan and Lumiracoxib enhance the interaction between STUB1 and TPIT, reducing POMC expression and ACTH secretion. **A** Schematic showing drug screening using the NanoBiT system. The NanoBiT assay was used to detect the interaction between Lgbit-STUB1 and Smbit-TPIT. A total of 1913 FDA-approved drugs were screened. **B** Interaction between STUB1 and TPIT. **C** Heatmap of candidate drugs influencing the interaction between STUB1 and TPIT. **D** The effects of Irbesartan, Bosentan, and Lumiracoxib on the STUB1-TPIT interaction were assessed by coimmunoprecipitation. **E** The effects of Irbesartan, Bosentan, and Lumiracoxib on TPIT ubiquitination were assessed by ubiquitination assays in HEK-293T cells. **F** Irbesartan and Lumiracoxib both reduced the protein levels of TPIT in AtT-20 cells while the proteasomal inhibitor MG132 (20 μM) reversed this effect. **G** Irbesartan and Lumiracoxib both suppressed *Pomc* in AtT-20 cells. Cells were treated with Irbesartan (1 μM) and Lumiracoxib (1 μM) and *Pomc* expression was measured by qPCR. Data are presented as mean ± SD values. n = 3. ***p < 0.001. **H** AtT-20 cells were treated with Irbesartan (1 μM) and Lumiracoxib (1 μM), and immunoblotted. **I** Irbesartan and Lumiracoxib inhibited the secretion of ACTH in AtT-20 cells, while knockdown of STUB1 partly reversed this effect. AtT-20 cells were treated with Irbesartan and Lumiracoxib. ACTH levels were measured by ELISA. Data are presented as mean ± SD values. n = 3. *p < 0.05, ***p < 0.001

Enhancing the interaction between STUB1 and TPIT has the potential to increase TPIT ubiquitination and degradation, offering a potential therapeutic approach for CD. To investigate potential drug candidates that can enhance this interaction, a NanoBiT system was developed to evaluate the binding between Lgbit-STUB1 and Smbit-TPIT, followed by a drug screening process involving 1913 FDA-approved compounds (Fig. 7A, B). A number of drugs were found to enhance or reduce the interaction between STUB1 and TPIT, and the top 10 enhancing drugs and 2 reducing drugs were selected for further verification (Fig. 7C). Co-IP assays showed that Irbesartan, Bosentan, and Lumiracoxib enhanced the interaction between STUB1 and TPIT (Fig. 7D). Moreover, Irbesartan and Lumiracoxib promoted the ubiquitination of TPIT, but Bosentan failed (Fig. 6E). Further investigation into the impact of Irbesartan and Lumiracoxib on TPIT, POMC, and ACTH levels revealed that both compounds decreased TPIT protein levels, which was reversed by the proteasomal inhibitor MG132 (Fig. 7F). Additionally, Irbesartan and Lumiracoxib suppressed *Pomc* transcription but did not affect *Tpit* and *Stub1* transcription in AtT-20 cells (Fig. 7G, Supplementary Fig. 6A, B). Furthermore, both Irbesartan and Lumiracoxib demonstrated a reduction in TPIT and POMC protein levels, while showing no impact on STUB1 (Fig. 7H). Subsequent evaluation of ACTH secretion using ELISA indicated that both Irbesartan and Lumiracoxib significantly suppressed ACTH secretion in AtT-20 cells. However, the knockdown of STUB1 partially mitigated this inhibitory effect (Fig. 7I). Cell toxicity analysis confirmed the non-toxic nature of Irbesartan and Lumiracoxib on AtT-20 cells (Supplementary Fig. 6A, B), suggesting that the inhibition of ACTH secretion by these compounds was not due to cell death. Furthermore, evidence suggests that Irbesartan and Lumiracoxib have the capability to directly bind to the STUB1 protein (Supplementary Fig. 8A, B). Moreover, these compounds have the potential to augment the efficacy of established therapies, such as Pasireotide (Supplementary Fig. 9A).

Collectively, these findings indicate that Irbesartan and Lumiracoxib facilitate the interaction between STUB1 and TPIT, leading to the degradation of TPIT, thereby decreasing POMC expression and ACTH secretion. Consequently, Irbesartan and Lumiracoxib hold promise as potential therapeutic agents for CD.

## Discussion

Ubiquitination of proteins plays a key role in regulating the stability and activity of various tumor-related proteins [5]. TPIT, as the main transcriptional activator of the *POMC* gene, is crucial for the occurrence and development of CD [27]. Our previous study identified the ubiquitin ligase TRIM65 for TPIT through a yeast two-hybrid technique [9]. This study utilized Co-IP and mass spectrometry techniques to investigate potential ubiquitin ligases and deubiquitinases for TPIT and identified two novel E3 ligases, STUB1 and TRIM28, which interact with TPIT to regulate its degradation. Further experiments found that STUB1 suppresses ACTH secretion by degrading TPIT and inhibits cell proliferation. Additionally, STUB1 was decreased in ACTH-secreting corticotroph adenoma compared to SCAs. STUB1 may become a potential therapeutic target for managing CD.

It is important to acknowledge a potential limitation regarding the transcriptomic data. The observed imbalance in cohort sizes between functional corticotroph adenomas and SCAs in our RNA-seq analysis may introduce bias into the identification of differential signatures. Larger, balanced multi-centre collections will be essential in future studies to confirm the generalizability and robustness of the identified transcriptomic profiles distinguishing these adenoma subtypes.

STUB1 is recognized as a tumor suppressor in liver, prostate, lung, and gastric cancers through the degradation of key oncogenic proteins, such as JMJD1A, YAP1, AR1, and YTHDF2 [13, 14, 28–30]. STUB1 is mainly localized cytoplasm and highly expressed in skeletal muscle, heart, pancreatic, and brain tissue [11, 31]. Notably, STUB1 possesses the ability to target cell surface, cytoplasmic, nuclear, or secreted proteins for ubiquitin-dependent degradation [24]. Studies have indicated that the loss of nuclear, but not cytoplasmic, STUB1 is associated with increased tumor aggressiveness and reduced survival in patients with breast cancer [32]. TPIT is mainly localized in the nucleus, and the staining in the current study revealed that STUB1 is expressed and localized in both the cytoplasm and nucleus. This study found that the nuclear-localized STUB1 was upregulated in SCAs and negatively correlated with the expression of *POMC*. JG98, a novel allosteric HSP70 inhibitor, and inflammation-associated stimulation can promote the nuclear translocation of STUB1 [33, 34]. In the future, drugs and immunotherapy could be explored to promote the nuclear translocation of STUB1, thereby enhancing its ubiquitination and degradation of TPIT.

The tumor size is usually that of a microadenoma, and there is no space-occupying effect in CD. Its harm is mainly the result of changes in the peripheral system caused by excessive secretion of ACTH. Therefore, we propose the therapeutic strategy of targeting the specific transcription factor TPIT, leading to inhibition of *POMC* transcription and reduced ACTH secretion, and thus controlling the harmful effects of CD. Pharmacological treatment of CD is usually aimed at suppressing cortisol production or blocking its action. Commonly used medications include dopamine agonists, somatostatin analogs, mifepristone, ketoconazole, and metopirone [35]. It is important to note that pharmacological treatment is usually long-term and may be associated with side effects, such as hypertension, edema, and electrolyte imbalances [36]. In contrast, the present study screened ubiquitin ligases to regulate the levels of TPIT, thus influencing the expression of *POMC* and the secretion of ACTH. Furthermore, two drugs approved by the Food and Drug Administration [37], Irbesartan and Lumiracoxib, were identified to promote the interaction between STUB1 and TPIT, leading to the degradation of TPIT, thereby downregulating *POMC* expression and ACTH secretion. While Irbesartan and Lumiracoxib show promise in promoting STUB1-mediated TPIT degradation, it is crucial to consider their known primary targets (e.g., angiotensin II receptor type 1 and COX-2, respectively) [38–40]. Our observation that STUB1 knockdown only partially reversed the inhibitory effect of these drugs on ACTH secretion suggests they may also act through additional, STUB1-independent pathways to suppress ACTH. The precise nature of these alternative mechanisms requires further exploration in future studies.

Irbesartan is an antihypertensive drug commonly used in clinical practice, while Lumiracoxib is a selective COX-2 inhibitor. Irbesartan has been studied and found to have no adverse effects on cancer risk, while also demonstrating promising potential in overcoming drug resistance in pancreatic cancer and activating the immune system in patients with colorectal cancer [38–40]. Lumiracoxib has demonstrated antiproliferative effects on human non-small cell lung cancer cell lines and may have the potential to reduce the risk of skin cancer [41, 42]. However, it is important to note that Lumiracoxib, along with several other drugs, has been withdrawn from the market due to its potential to cause liver damage [43]. Therefore, further investigation from a structural biology perspective is warranted, together with in vivo studies on animal models to evaluate the efficacy of these drugs and lay the groundwork for pre-clinical trials. Beyond its role in suppressing ACTH secretion via TPIT degradation, our data also demonstrates that STUB1 inhibits corticotroph tumor cell proliferation. STUB1 is likely to suppress AtT-20 growth through targets other than TPIT. Identifying additional STUB1 substrates relevant to corticotroph tumor growth is an important avenue for future research. Furthermore, the use of the proteolysis-targeting chimera (PROTAC) technique to degrade proteins crucial for tumor development has emerged as a potential cancer treatment strategy [44, 45]. TPIT as a pituitary-specific transcription factor and the identification of its ubiquitin ligases provides the foundation for the development of PROTAC drugs.

In summary, as TPIT is a key regulatory factor in the occurrence and development of CD, regulation of its stability and activity through the ubiquitination pathway may provide new targets for tumor treatment. The study discovered a novel mechanism of STUB1-mediated TPIT ubiquitination and degradation and preliminarily verified the therapeutic potential of targeting the STUB1/TPIT interaction. In addition, exploring the use of immunotherapy and other means to promote the redistribution of STUB1 in cells and enhance its ubiquitination of TPIT is also a novel direction worthy of attention. In the future, in-depth research on the ubiquitination-mediated regulation of TPIT and the development of more efficient and specific small-molecule regulators will provide new avenues for the precise treatment of CD.

## Conclusion

In this study, we discovered that a novel E3 ubiquitin ligase STUB1 interacts with transcription factor TPIT, leading to proteasomal degradation of TPIT. Moreover, STUB1 could inhibit POMC expression, ATCH secretion and cell proliferation in AtT-20 cells. Additionally, STUB1 was decreased in ACTH-secreting corticotroph adenoma compared to SCAs and negatively correlated with TPIT protein, as well as expression of POMC. Furthermore, drug screening identified the Irbesartan and Lumiracoxib promoted the interaction between STUB1 and TPIT, leading to proteasomal degradation of TPIT and the suppression of ACTH secretion.

## Supplementary Information

Supplementary Material 1

Supplementary Material 2

Supplementary Material 3

Supplementary Material 4

Supplementary Material 5

Supplementary Material 6

Supplementary Material 7

Supplementary Material 8

Supplementary Material 9

Supplementary Material 10

Supplementary Material 11

## Data Availability

The raw sequence data reported in this paper have been deposited in the Genome Sequence Archive [46] in National Genomics Data Center [47], China National Center for Bioinformation / Beijing Institute of Genomics, Chinese Academy of Sciences (GSA-Human: HRA005096) that are publicly accessible at https://ngdc.cncb.ac.cn/gsa-human.
