# Supplementary material for: The STUB1-TPIT axis regulates the secretion of adrenocorticotrophic hormone in cushing disease
Source: J Transl Med. 2025 Aug 26;23:961. doi: 10.1186/s12967-025-06960-y (PMC12382147; doi:10.1186/s12967-025-06960-y)
Supplement: Supplementary file 1 — Supplementary Material 1 [file 12967_2025_6960_MOESM1_ESM.docx]

**Supplementary Materials**

**Supplementary Figure 1** **STUB1, TRIM28, and PLRG1 interact with TPIT. (A)** Interactions between TPIT and candidate ubiquitin ligases in HEK-293T cells were assessed by coimmuniprecipitation. Except for the HUWE1 protein carrying a FLAG tag, all other co-IP experiments employed TPIT fused to FLAG. FLAG-tagged proteins were consistently used as the bait to assess protein interactions. **(B)** STUB1 and TRIM28 promoted the ubiquitination of TPIT. HEK-293T cells were transfected with indicated plasmids followed by ubiquitination assays. **(C)** STUB1 and TRIM28 both reduced the protein levels of Flag-TPIT in HEK-293T cells. Stably transfected HEK-293T cells expressing Flag-TPIT were transfected with the indicated plasmids before immunoblotting.

**Supplementary Figure 2 STUB1 is mainly localized in the cytoplasm in HEK-293T cells. (A-B)** qPCR validation of STUB1 overexpression or knockdown efficiency in HEK293T. Data are presented as mean ± SD values. n = 3. ****p* < 0.001. **(C)** Localization of Myc-STUB1 in HEK-293T cells. Scale bar, 5 μm.

**Supplementary Figure 3 *Stub1* expression has no effect on the mRNA expression of *Tpit*. (A-B)** qPCR validation of STUB1 overexpression or knockdown efficiency in AtT-20. Data are presented as mean ± SD values. n = 3. ****p* < 0.001. **(C-D)** qPCR showed that overexpression (A) or knockdown (B) of *Stub1* had no effect on the mRNA expression of *Tpit* in AtT-20 cells. Data are presented as mean ± SD values. n = 3. n.s., not significant.

**Supplementary Figure 4 STUB1 regulates cell proliferation in AtT-20 cells. (A-B)** *Stub1* inhibited proliferation of AtT20 cells. Stably transfected AtT-20 cells with *Stub1* overexpression (A) or knockdown (B) were assessed by luminescent cell viability assays. Data are presented as mean ± SD values. n = 3. ****p* < 0.001.

**Supplementary Figure 5 *STUB1* is downregulated in corticotroph adenomas. (A)** *STUB1* is downregulated in corticotroph adenomas. Bulk RNA sequencing was conducted on corticotroph adenomas (n = 56) compared to normal pituitary tissues (n = 107). Data are presented as mean ± SD values. ****p* < 0.001. **(B)** Correlation between the mRNA expression of *STUB1* and *TPIT* in corticotroph adenomas. **(C)** Correlation between nuclear-localized STUB1 and mRNA expression of *TPIT* in corticotroph adenomas.

**Supplementary Figure 6 Irbesartan and Lumiracoxib have no effect on mRNA expression of *Tpit* and *Stub1*. (A-B)** qPCR showed that Irbesartan and Lumiracoxib had no effect on mRNA expression of *Tpit* (A) and *Stub1* (B) in AtT-20 cells. Data are presented as mean ± SD values. n = 3. n.s., not significant.

**Supplementary Figure 7 Irbesartan and Lumiracoxib are non-toxic to AtT-20 cells. (A-B)** AtT-20 cells were treated with varying concentrations of Irbesartan and Lumiracoxib for 24 h or 48 h and cell viability was measured by luminescent cell viability assays. Data are presented as mean ± SD values. n = 3.

**Supplementary Figure 8 Irbesartan and Lumiracoxib could directly bind to STUB1.**

**(A-B)** STUB1 proteins were immobilized on a CM5 chip as the solid phase. Irbesartan (A) and Lumiracoxib (B) were subsequently serially diluted to generate concentration gradients, facilitating their interaction with the immobilized protein.

**Supplementary Figure 9 Irbesartan and Lumiracoxib synergize with pasireotide to suppress ACTH secretion.**

**(A-B)** AtT-20 cells were treated with Irbesartan (1 μM), Lumiracoxib (1 μM) and pasireotide (10 nM). ACTH levels were measured by ELISA. Data are presented as mean ± SD values. n = 3. ****p* < 0.001.
