# Supplementary figures and images for: The STUB1-TPIT axis regulates the secretion of adrenocorticotrophic hormone in cushing disease
Source: J Transl Med. 2025 Aug 26;23:961. doi: 10.1186/s12967-025-06960-y (PMC12382147; doi:10.1186/s12967-025-06960-y)

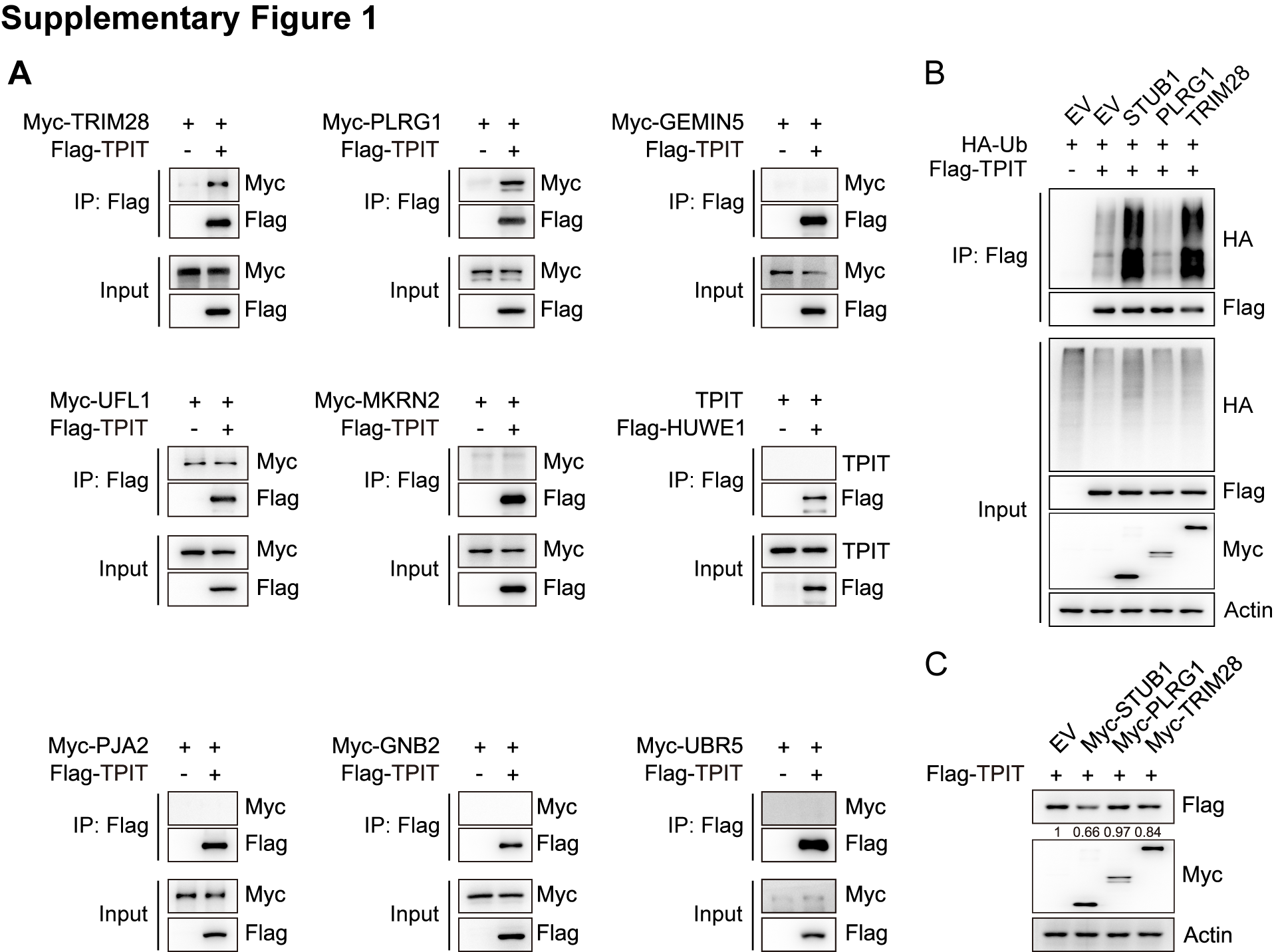

Supplement: Supplementary file 2 — Supplementary Material 2 [file 12967_2025_6960_MOESM2_ESM.tif]

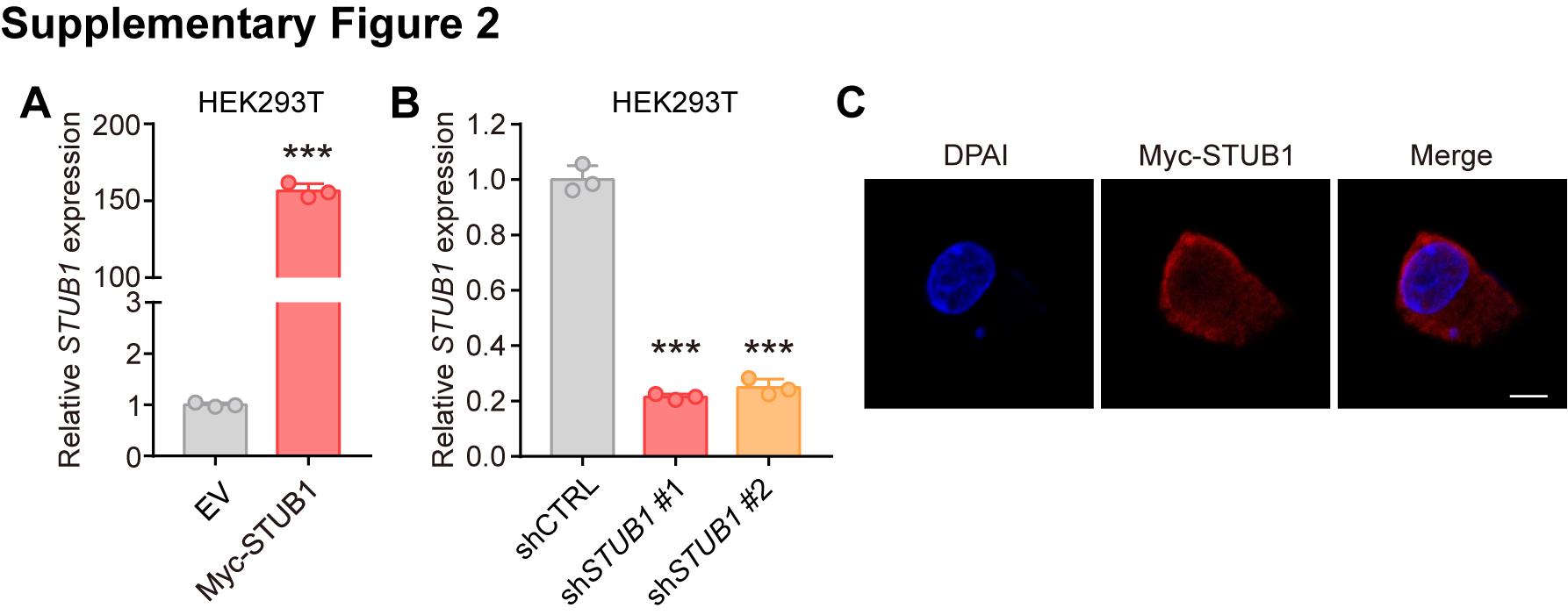

Supplement: Supplementary file 3 — Supplementary Material 3 [file 12967_2025_6960_MOESM3_ESM.tif]

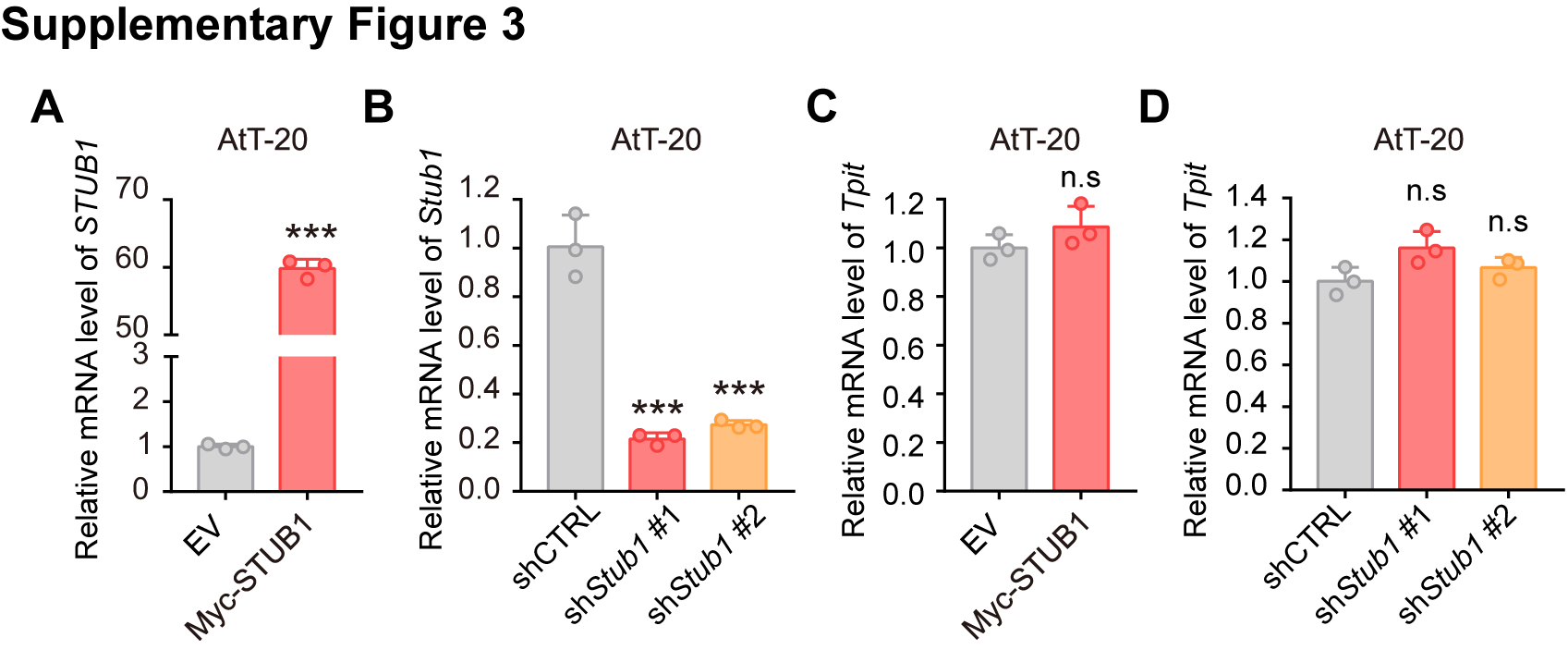

Supplement: Supplementary file 4 — Supplementary Material 4 [file 12967_2025_6960_MOESM4_ESM.tif]

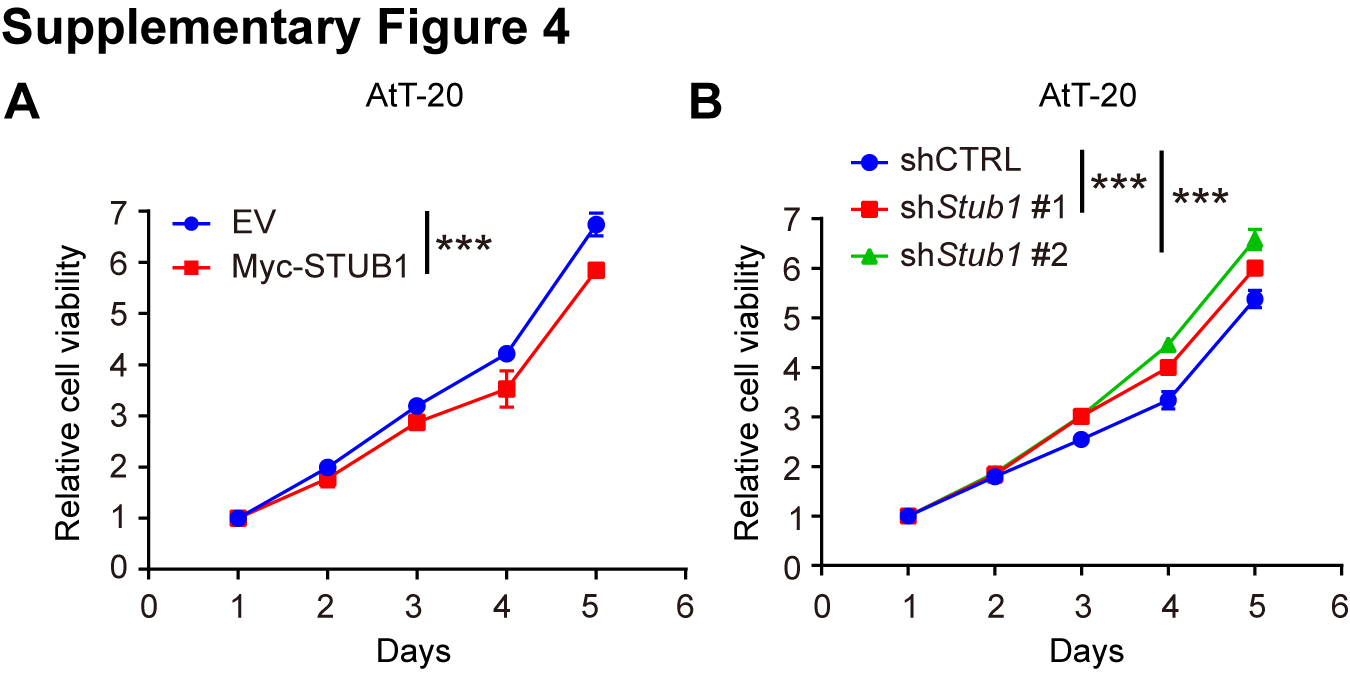

Supplement: Supplementary file 5 — Supplementary Material 5 [file 12967_2025_6960_MOESM5_ESM.tif]

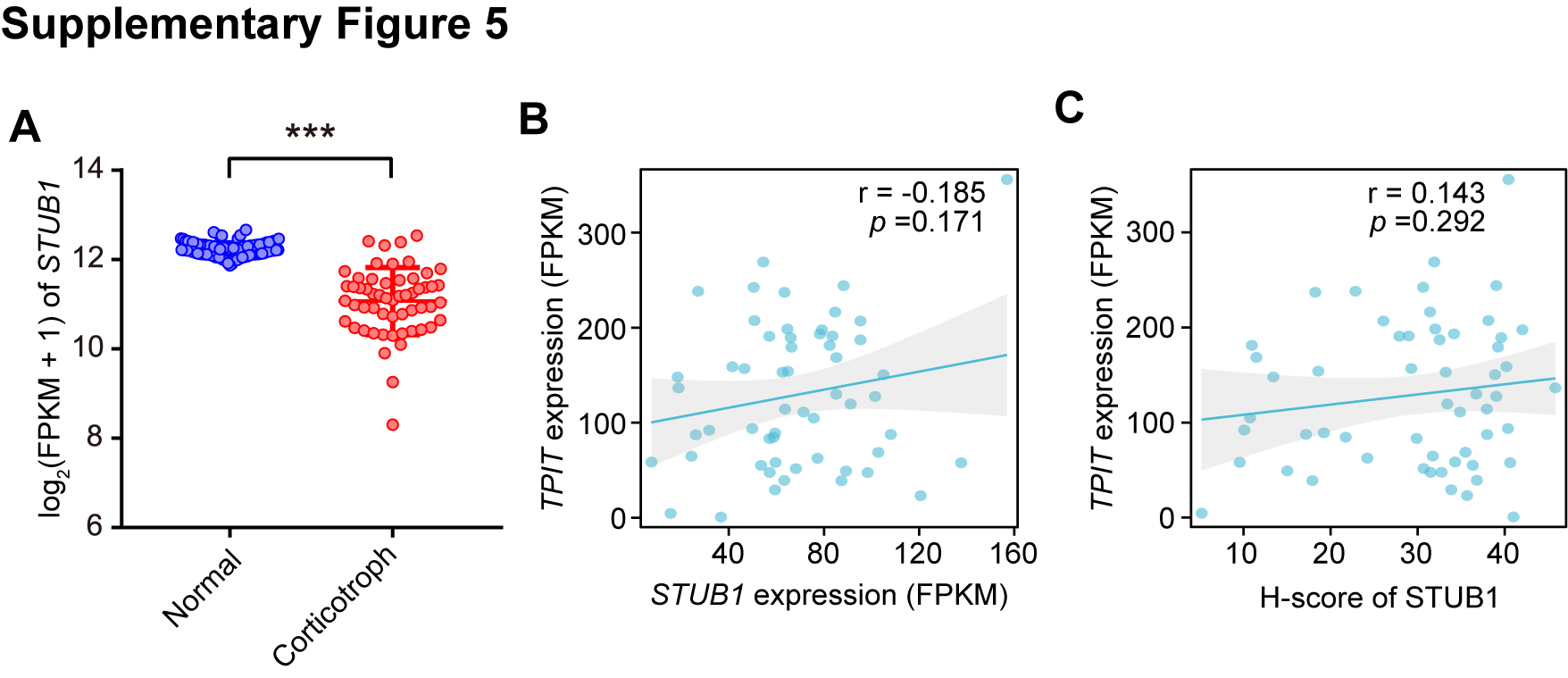

Supplement: Supplementary file 6 — Supplementary Material 6 [file 12967_2025_6960_MOESM6_ESM.tif]

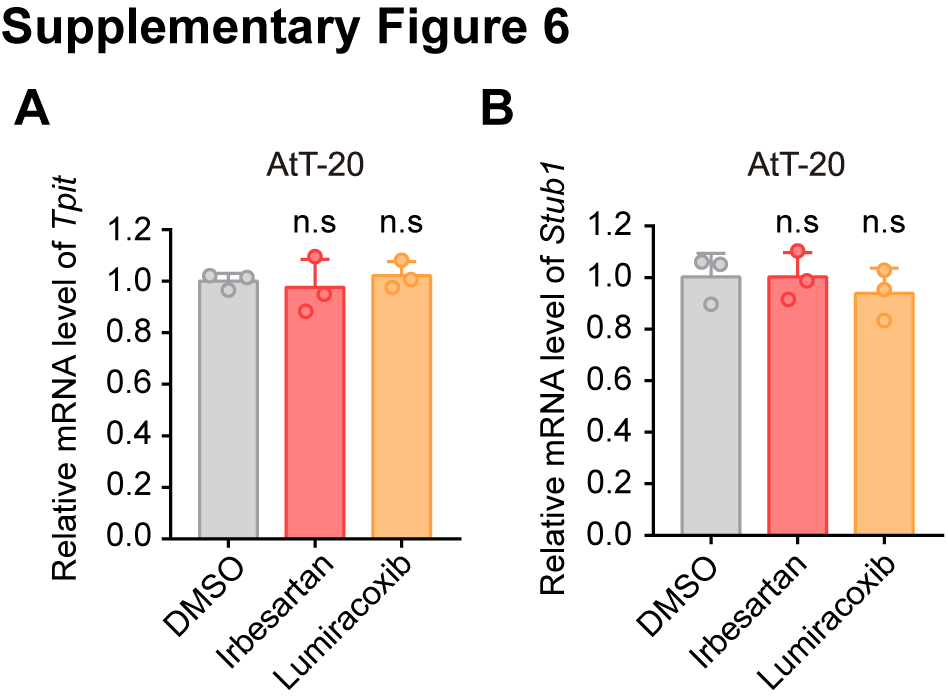

Supplement: Supplementary file 7 — Supplementary Material 7 [file 12967_2025_6960_MOESM7_ESM.tif]

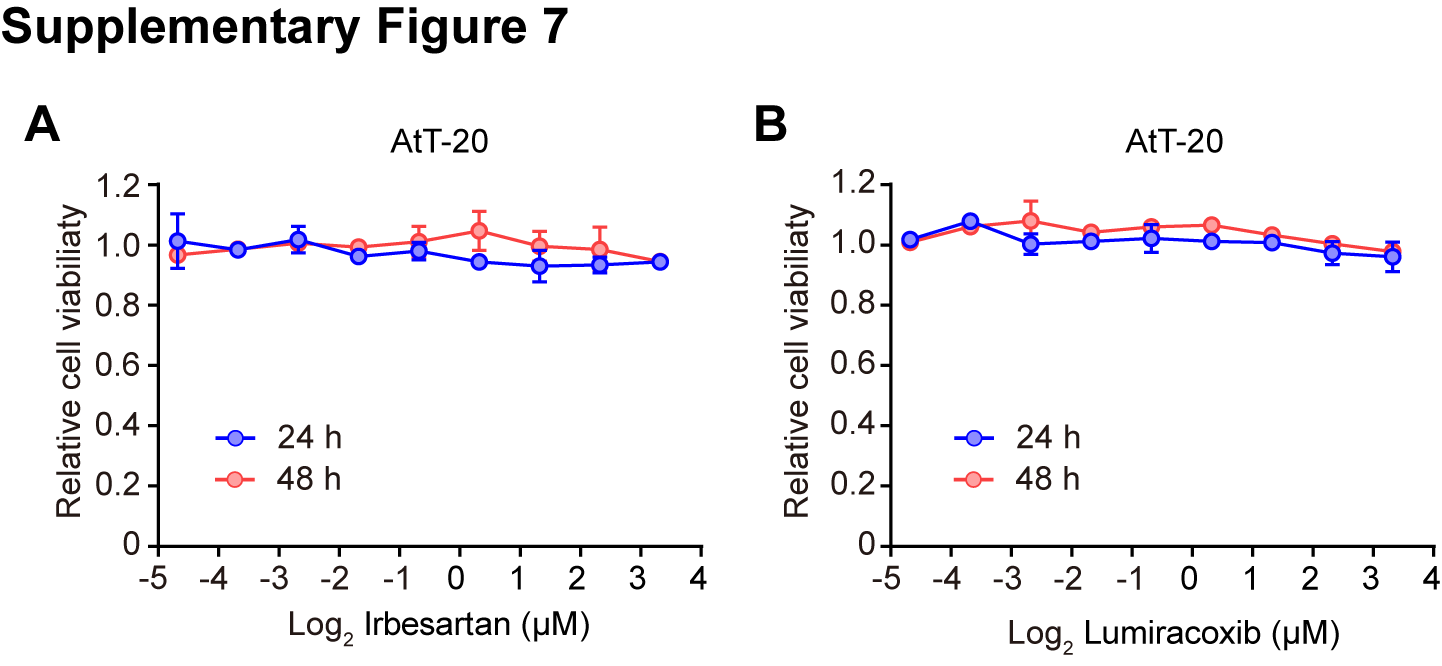

Supplement: Supplementary file 8 — Supplementary Material 8 [file 12967_2025_6960_MOESM8_ESM.tif]

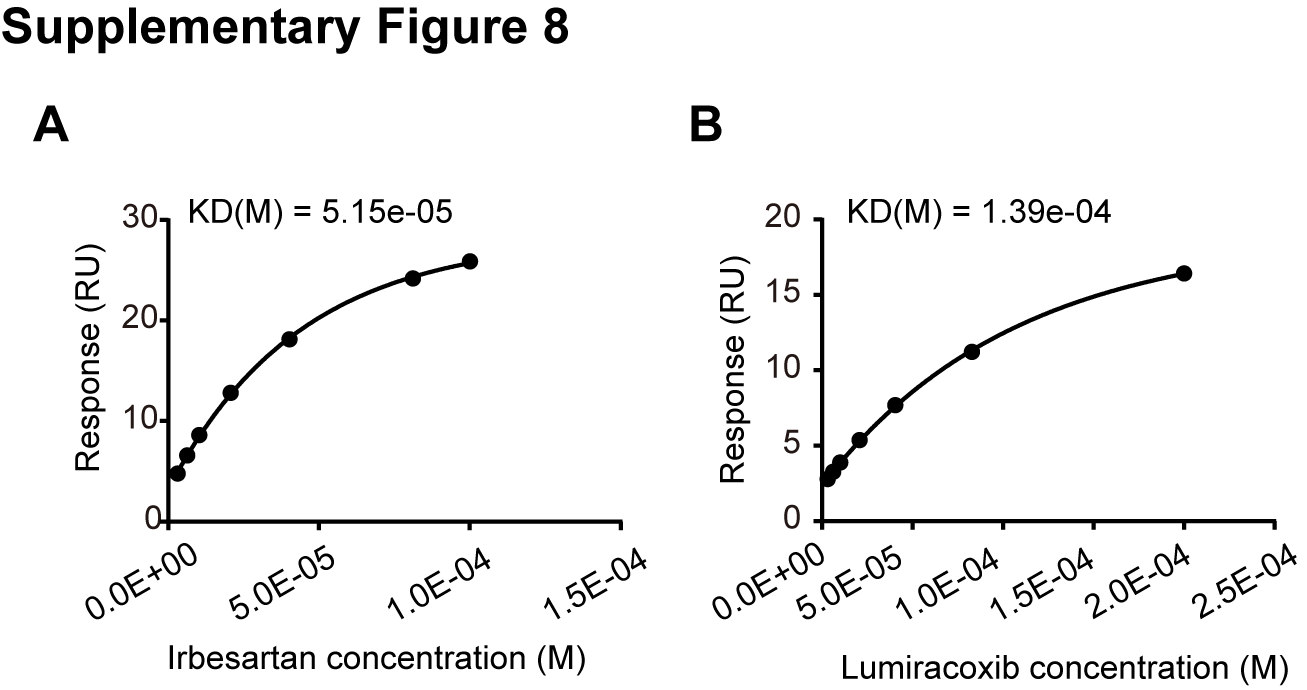

Supplement: Supplementary file 9 — Supplementary Material 9 [file 12967_2025_6960_MOESM9_ESM.tif]

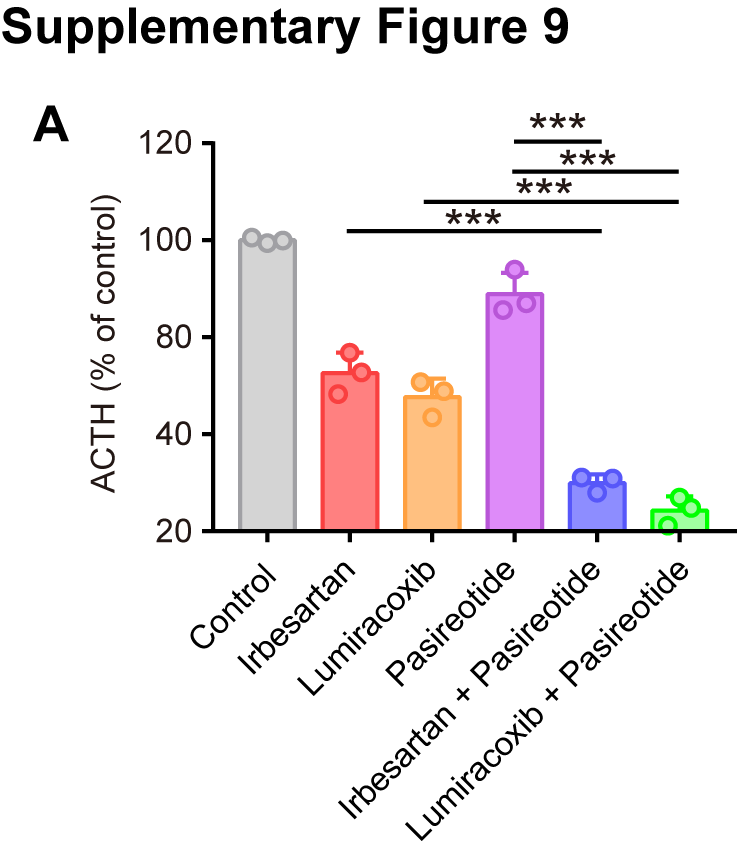

Supplement: Supplementary file 10 — Supplementary Material 10 [file 12967_2025_6960_MOESM10_ESM.tif]
